# Gastric microbial community profiling reveals a dysbiotic cancer-associated microbiota

**DOI:** 10.1136/gutjnl-2017-314205

**Published:** 2017-11-04

**Authors:** Rui M Ferreira, Joana Pereira-Marques, Ines Pinto-Ribeiro, Jose L Costa, Fatima Carneiro, Jose C Machado, Ceu Figueiredo

**Affiliations:** 1 i3S – Instituto de Investigação e Inovação em Saúde, Universidade do Porto, Porto, Portugal; 2 Ipatimup – Institute of Molecular Pathology and Immunology of the University of Porto, Porto, Portugal; 3 Institute of Biomedical Sciences Abel Salazar (ICBAS), University of Porto, Porto, Portugal; 4 Faculty of Medicine, University of Porto, Porto, Portugal; 5 Department of Pathology, Centro Hospitalar São João, Porto, Portugal

**Keywords:** gastric carcinoma, gastritis, *Helicobacter pylori*, bacterial infection

## Abstract

**Objective:**

Gastric carcinoma development is triggered by *Helicobacter pylori*. Chronic *H. pylori* infection leads to reduced acid secretion, which may allow the growth of a different gastric bacterial community. This change in the microbiome may increase aggression to the gastric mucosa and contribute to malignancy. Our aim was to evaluate the composition of the gastric microbiota in chronic gastritis and in gastric carcinoma.

**Design:**

The gastric microbiota was retrospectively investigated in 54 patients with gastric carcinoma and 81 patients with chronic gastritis by 16S rRNA gene profiling, using next-generation sequencing. Differences in microbial composition of the two patient groups were assessed using linear discriminant analysis effect size. Associations between the most relevant taxa and clinical diagnosis were validated by real-time quantitative PCR. Predictive functional profiling of microbial communities was obtained with PICRUSt.

**Results:**

The gastric carcinoma microbiota was characterised by reduced microbial diversity, by decreased abundance of *Helicobacter* and by the enrichment of other bacterial genera, mostly represented by intestinal commensals. The combination of these taxa into a microbial dysbiosis index revealed that dysbiosis has excellent capacity to discriminate between gastritis and gastric carcinoma. Analysis of the functional features of the microbiota was compatible with the presence of a nitrosating microbial community in carcinoma. The major observations were confirmed in validation cohorts from different geographic origins.

**Conclusions:**

Detailed analysis of the gastric microbiota revealed for the first time that patients with gastric carcinoma exhibit a dysbiotic microbial community with genotoxic potential, which is distinct from that of patients with chronic gastritis.

**Significance of this study**

**What is already known on this subject?**

- *Helicobacter pylori* infection increases the risk for gastric carcinoma by causing chronic inflammation and decreasing the number of acid-producing glands.
- Gastric acid reduction by acid-suppressive drugs results in bacterial overgrowth and high levels of gastric nitrite and *N*-nitrosamine.
- The context of a complex microbiota accelerates the onset and promotes neoplasia in the *H. pylori* insulin-gastrin mouse model of gastric cancer.

**What are the new findings?**

- The gastric microbiota profile of patients with carcinoma is significantly different from that of patients with chronic gastritis.
- The gastric carcinoma microbiota is dysbiotic and characterised by reduced microbial diversity, reduced *Helicobacter* abundance and over-representation of bacterial genera that include intestinal commensals.
- The microbial community found in gastric carcinoma has increased nitrosating functions consistent with increased genotoxic potential.

**How might it impact on clinical practice in the foreseeable future?**

Our results provide a new interpretative frame for understanding the microbial dysbiosis associated with gastric carcinoma, and suggest that alterations in the gastric microbiota may need to be considered to maximise efficacy of preventive and therapeutic strategies tailored at reducing the incidence of gastric carcinoma.

## Introduction

Gastric carcinoma is a major health problem worldwide, with an estimated 1 million new cases every year.^1^ *Helicobacter pylori* infection plays a crucial role in the initial steps of carcinogenesis by causing enhanced inflammation and progressive degradation of the architecture and function of the gastric epithelium.^2 3^ From a certain point on, however, gastric carcinoma development may be *H. pylori* independent, since colonisation decreases (and is eventually lost) in later steps of carcinogenesis.^4^ Additionally, *H. pylori* eradication studies have shown that successful eradication does not completely prevent gastric carcinoma development.^5–7^ These observations suggest that factors other than *H. pylori* contribute to persistent inflammation of the gastric mucosa and to gastric cancer development.

It has been proposed that changes that occur in the stomach as a result of chronic *H. pylori* infection leading to decreased acid secretion allow the successful establishment of a new microbiota that contributes to malignant transformation through maintenance of inflammation and conversion of nitrates into *N*-nitrosamines.^2 3^ This is supported by earlier studies showing that reduction of gastric acid by different types of drugs results in significant intragastric bacterial overgrowth, increased counts of nitrate-reducing bacteria and increased nitrite and *N*-nitrosamine levels.^8 9^ This is further supported by studies in the hypergastrinaemic insulin-gastrin (INS-GAS) transgenic mouse model, which showed that *H. pylori-*induced gastric cancer is promoted by the presence of a complex gastric microbiota, as these animals develop more tumours than germ-free mice infected with *H. pylori* only.^10 11^

So far, only a very small number of studies characterised the human gastric microbiota in health and disease. Their major findings were that *H. pylori*-negative subjects contained a diverse microbiota in their stomach, whereas in *H. pylori-*infected patients the gastric mucosa was dominated by this species.^12–15^ In the context of gastric carcinogenesis, few studies have been conducted and no particular component of the microbiota has been identified as implicated in gastric carcinoma.^15 16^ The limitations of these studies are the limitations in sensitivity and coverage compared with more recently developed techniques, and, most importantly, the inclusion of very limited numbers of subjects, making it difficult to generate statistically significant conclusions. Therefore, we performed high-throughput profiling of the gastric bacterial communities present in 135 gastric carcinoma cases and chronic gastritis controls, by next-generation sequencing (NGS) of the 16S rRNA gene. We used validation cohorts from multiple geographic locations to confirm our findings.

## Materials and methods

### Patients

Eighty-one individuals with chronic gastritis and 54 with gastric carcinoma were included in the Portuguese discovery cohort (see online supplementary table S1). These were part of a case–control study aimed at investigating risk modifiers for gastric cancer.^17 18^ Subjects with chronic gastritis (mean age 43.6±7.0 years; male-to-female ratio 39.5:1) were recruited during a screening programme for premalignant lesions of the gastric mucosa and underwent standard gastroscopy at Centro Hospitalar São João (CHSJ). Eleven patients presented glandular atrophy with foci of intestinal metaplasia. Of these, 1 had mild corpus and moderate antral atrophy and the remaining 10 cases did not have corpus atrophy and had mild (n=6), moderate (n=2) or marked (n=2) atrophy in the antrum (including incisura). Only individuals without evidence of past or present peptic ulcer disease were included. In addition, patients under proton pump inhibitor or antimicrobial treatments were excluded. Patients with gastric carcinoma (mean age 58.8±13.2 years; male-to-female ratio 1.5:1) were diagnosed and underwent cancer resection at CHSJ. A validation cohort of an additional 38 gastric specimens from 15 patients with chronic gastritis and 23 patients with gastric carcinoma, diagnosed between 2014 and 2016, were retrieved from the tissue and tumour bank at CHSJ (see online supplementary table S1). All procedures were in accordance with the institutional ethical standards. Samples were delinked and unidentified from their donors.

10.1136/gutjnl-2017-314205.supp1 Supplementary file 1

Two additional validation series, consisting of NGS data of the 16S rRNA gene of 79 gastric carcinoma cases from a population from China and 53 gastric carcinoma cases from a population from Mexico, were retrieved from the Sequence Read Archive (BioProject PRJNA310127; see online supplementary table S2).^19^

### 16S rRNA gene sequencing

DNA was isolated from gastric biopsies or surgical specimens of non-neoplastic gastric mucosa adjacent to the tumour, as previously described.^17^ The 16S rRNA gene was amplified using primers U789F 5′-TAGATACCCTGGTAGTCC-3′ and U1053R 5′-CTGACGACAGCCATGC-3′ targeting the V5-V6 hypervariable regions and sequenced in an Ion PGM Torrent platform following manufacturer’s instructions. Primers were designed following recommendations reported by Andersson *et al*, and were extensively analysed using PrimerProspector (see online supplementary figure S1).^20 21^

### Sequencing data analysis

The performance of the UPARSE pipeline was evaluated and compared with that reported for samples of the Human Microbiome Project (HMP) data set.^22^ Using UPARSE (usearch_v7.0.1090_i86linux64), reads were filtered by imposing a maximum number of expected errors of 0.5 and a global trimming at 250 nucleotides.^22^ Reads were dereplicated and singletons were discarded. Filtered reads were clustered into operational taxonomic units (OTU) assuming 97% similarity. Chimeric reads were reference removed using Uchime.^22^ Each OTU was taxonomically assigned using Uclust considering a minimum percentage of similarity to a reference database (Greengenes Named Isolate database, release August 2013) match of 90%.^23^ Diversity analyses were performed using QIIME (V.1.9).^24^ Alpha diversity was determined by the Shannon index and with Good’s estimator of coverage. Differences in alpha diversity were assessed by the t-test controlled with 10^3^ Monte Carlo permutations. Beta diversity was assessed by unweighted and weighted UniFrac distance matrices and visualised by principal coordinate analysis (PCoA), controlled by 10^3^ jackknife replicates.^25^ Sample clustering in beta diversity analysis was tested using analysis of similarity (ANOSIM) with 10^4^ bootstrap replications.^26^ Comparisons between distance matrices were evaluated by the Mantel correlation controlled with 10^4^ permutations.

### Taxonomic discovery analysis

Statistically significant differences in the relative abundance of taxa associated with groups of patients were performed using linear discriminant analysis (LDA) effect size (LEfSe).^27^ Only taxa with LDA greater than 4 at a *P* value <0.05 were considered significantly enriched.

### Real-time quantitative PCR

Sequencing results were confirmed by quantitative PCR (qPCR) (see online supplementary table S3).

### Functional metagenome predictions

For functional metagenome prediction, we captured OTU representative sequences from Greengenes database using the USEARCH global alignment command and discarding reads that did not hit the reference database. Reconstruction of the metagenome was performed using PICRUSt.^28^ Accuracy of the predicted metagenomes was assessed by determining the nearest sequenced taxon index. Predicted functional genes were categorised into Clusters of Orthologous Groups (COG) and into Kyoto Encyclopedia of Genes and Genome (KEGG) orthology (KO), and compared across patient groups using STAMP.^29^ Statistical differences in COG and KO frequencies were determined by White’s non-parametric t-test with a Benjamini-Hochberg false discovery rate correction to adjust P values for multiple testing.^30^

For further details, see online supplementary information.

## Results

### Quality control of 16S rRNA microbiota profiling

In the present study, we compared the gastric microbiota of patients with chronic gastritis with that of patients with gastric carcinoma by NGS of the 16S rRNA gene. After sequencing and quality filtering, more than 10.8 million reads were obtained corresponding to a mean of 80 261 reads and 178 OTUs per sample (see online supplementary figure S2). On average, patients with chronic gastritis had a significantly higher number of reads (86 957) than patients with cancer (67 954; *P*<0.05). However, the number of OTUs was not significantly different between the two patient groups (186 and 169 OTUs, respectively; *P*=0.071). To control for the number of false OTUs and to measure the number of biologically meaningful OTUs, we classified them according to similarity shared with sequences of the Greengenes Named Isolated database (see online supplementary figure S2). In our data set, the frequencies of misleading and valid OTUs were similar to those reported for the HMP data set processed with the UPARSE pipeline.^22^

To assure consistency between amplification and sequencing sets, 32 randomly selected samples were used to test reproducibility. The intraclass correlation coefficients showed good reproducibility for the assessment of the Shannon index, for the UniFrac distances and for the relative abundance of phyla (see online supplementary table S4). In conclusion, our approach provides the most in-depth characterisation of the gastric microbiota so far and generates robust and consistent data.

### The gastric microbiota profile differs in chronic gastritis and gastric carcinoma

**Figure 1:**
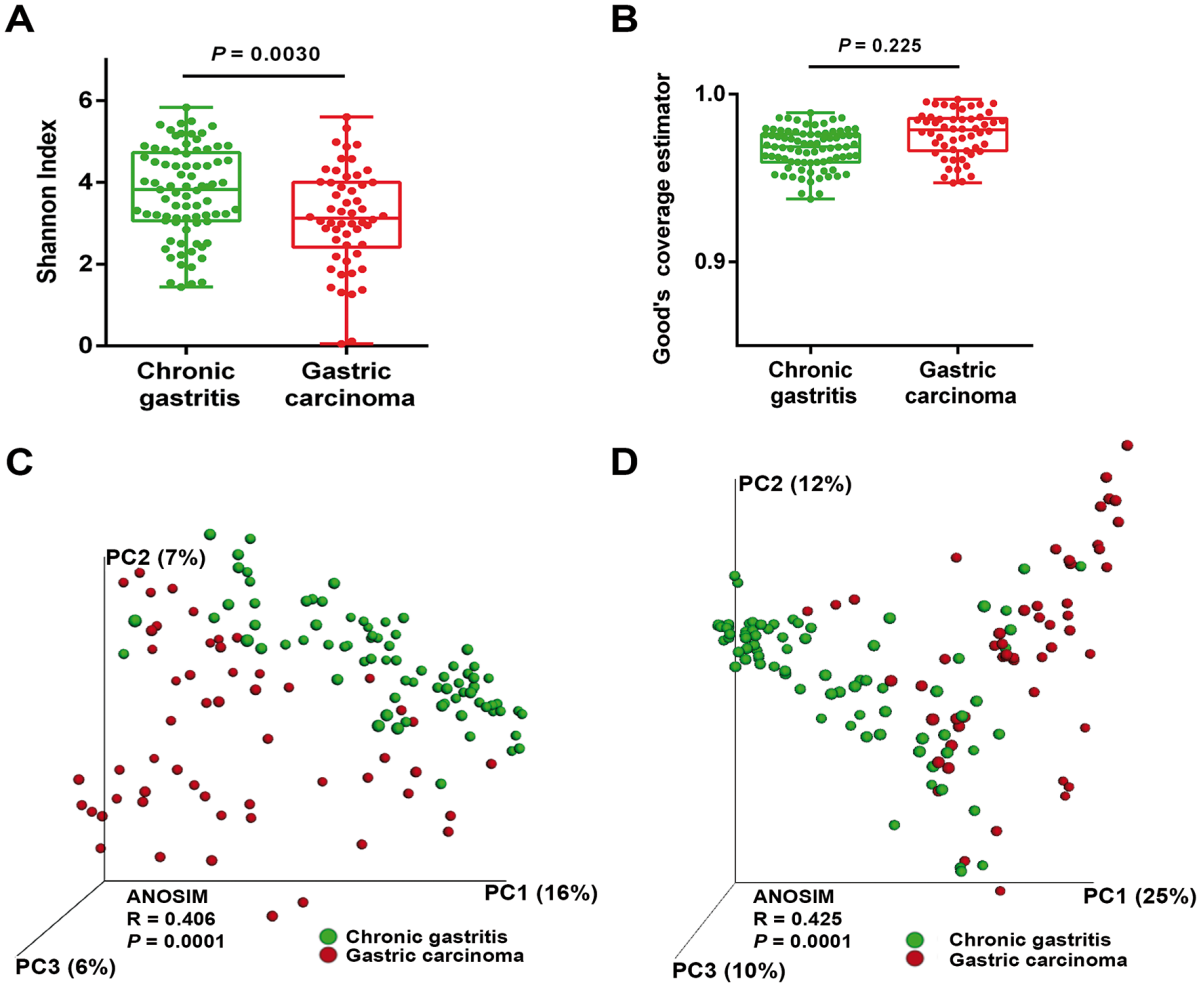
The gastric microbiota profile differs in chronic gastritis and gastric carcinoma. (A) Shannon index of diversity in patients with chronic gastritis and gastric carcinoma. (B) Good’s estimator of coverage, measuring the proportion of total bacterial species represented in samples of each group of patients. Principal coordinate analysis (PCoA) plots of (C) unweighted and (D) weighted UniFrac distances in which samples were coloured by clinical outcome. The percentage of diversity captured by each coordinate is shown. ANOSIM, analysis of similarity.

To evaluate alterations in the microbiota structure between patients with chronic gastritis and gastric carcinoma, we measured microbial alpha diversity (ie, within sample diversity) and beta diversity (ie, diversity between samples). By measuring alpha diversity using the Shannon index, we found that patients with gastric carcinoma had significantly decreased microbial diversity in comparison with patients with chronic gastritis (figure 1A, *P*=0.003; online supplementary figure S3). To ensure good estimation of bacterial diversity, we measured the proportion of total bacterial species represented in samples of each patient group by the Good’s estimator of coverage. Estimated coverage ranged from 0.94 to 0.98 in chronic gastritis and from 0.95 to 0.99 in gastric carcinoma (*P*=0.225), suggesting that the 16S rRNA results from each (chronic gastritis and gastric carcinoma) library represent the majority of bacteria present in the gastric mucosa (figure 1B).

Beta diversity was calculated using both unweighted (ie, qualitative) and weighted (ie, quantitative) UniFrac phylogenetic distance matrices, and visualised in PCoA plots. The total diversity captured by the top three principal coordinates was 29% and 47% for unweighted and weighted UniFrac, respectively. The microbiota composition of patients with gastric carcinoma was significantly different from that of patients with chronic gastritis (ANOSIM R=0.406, *P*=0.0001; and R=0.425, *P*=0.0001, for unweighted and weighted distances, respectively; figure 1C,D).

Since age is an established risk factor for gastric carcinoma, and since patients with carcinoma were significantly older than patients with gastritis in our series (see online supplementary table S1), we next addressed whether the microbial profile was different between younger and older patients. Overall, increasing age could differentiate the microbiota profiles of the full sample set (see online supplementary figure S4A,B). However, when we performed age-matched comparisons of the microbiota in patients with chronic gastritis and carcinoma, we observed statistically significant differences in the unweighted and weighted UniFrac distances (see online supplementary figure S4C,D), reinforcing that the microbiota composition is different in the two clinical settings. Also in the age-matched comparisons, significantly decreased microbial alpha diversity was found in patients with gastric carcinoma (see online supplementary figure S4E, *P*=0.0096).

No statistically significant differences in the microbiota profiles of gastric carcinoma cases were observed for gender, histological type and tumour location (see online supplementary figure S5).

In patients with chronic gastritis, we could not detect differences in the alpha diversity and beta diversity between patients with non-atrophic gastritis (n=70) and patients with glandular atrophy (n=11; online supplementary figure S6), and therefore they were pooled together for the analyses.

These results show that there is a significant reduction in microbial diversity in gastric carcinoma. Furthermore, the fact that the weighted UniFrac captured more diversity than unweighted metrics suggests that alterations in the relative abundance of taxa are a major contributor for microbiota differences between gastritis and gastric carcinoma.

### The influence of *H. pylori* in the microbiota composition of chronic gastritis and gastric carcinoma

**Figure 2:**
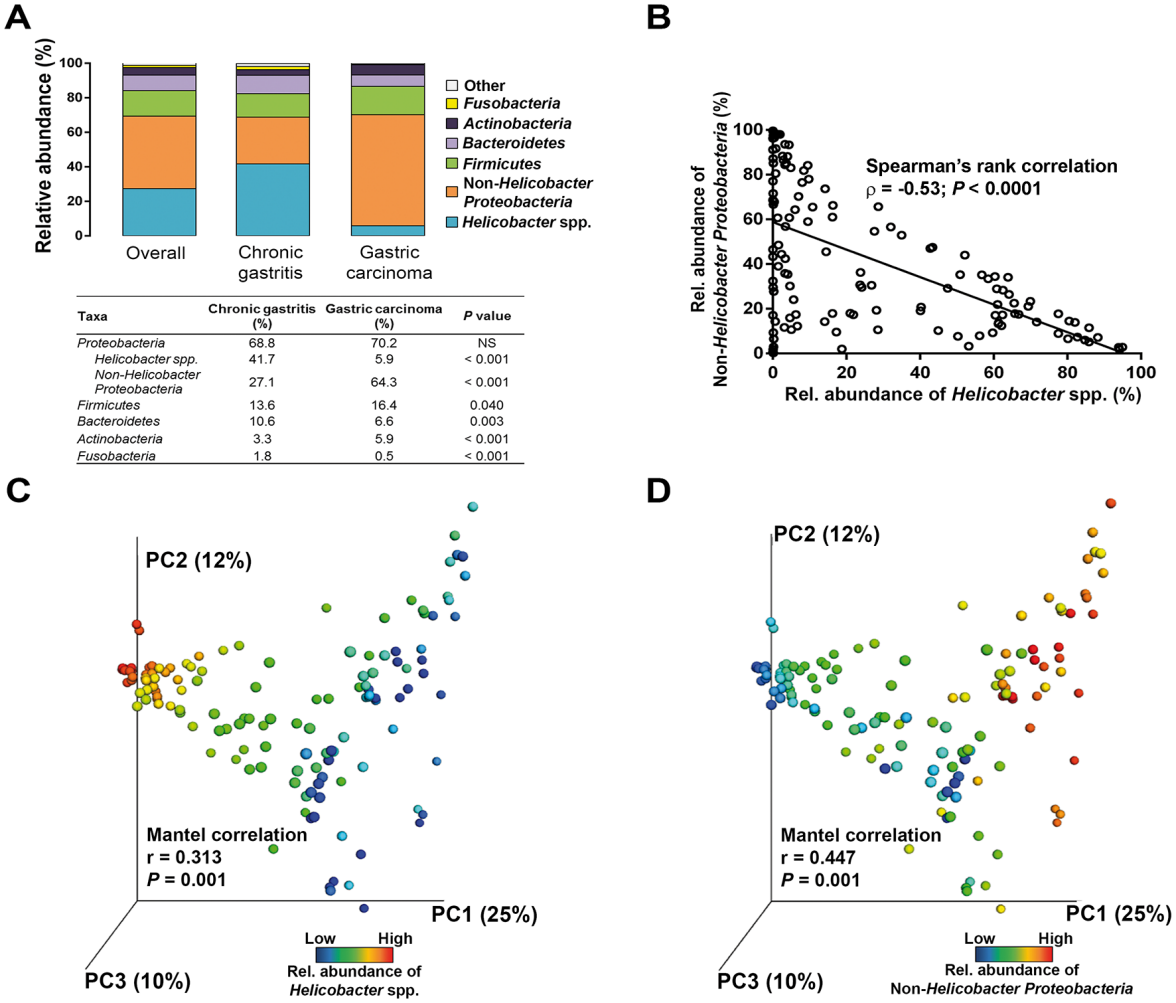
The influence of *Helicobacter pylori* in the microbiota composition of chronic gastritis and gastric carcinoma. (A) Relative abundance of phyla in all subjects and in each group of patients. (B) Spearman’s rank correlation between relative abundance of *Helicobacter* spp. and non-*Helicobacter Proteobacteria* in all patients. Principal coordinate analysis (PCoA) plots of the weighted UniFrac distance matrix coloured by (C) increasing relative abundance of *Helicobacter* and of (D) non-*Helicobacter Proteobacteria*.

Overall, the gastric microbiota was dominated by five phyla: *Proteobacteria* (69.3%), *Firmicutes* (14.7%), *Bacteroidetes* (9.0%), *Actinobacteria* (4.3%) and *Fusobacteria* (1.3%). Although these phyla were present in the two patient groups in the same order of relative abundance, the gastric carcinoma microbiota had an over-representation of *Actinobacteria* (P<0.001) and *Firmicutes* (P=0.040), and lower abundance of *Bacteroidetes* (*P*=0.003) and *Fusobacteria* (*P*<0.001; figure 2A).

When reads assigned to *Proteobacteria* into *Helicobacter* spp. and non-*Helicobacter Proteobacteria* were separated, a significant reduction in the abundance of *Helicobacter* (*P*<0.001) and an over-representation of non-*Helicobacter Proteobacteria* were detected in gastric carcinoma (*P*<0.001; figure 2A). Accordingly, a significant negative correlation was found between these taxa (r=−0.53, *P*<0.0001; figure 2B). In support of the above, the microbiota profile of the two patient groups could be distinguished by the abundance of *Helicobacter* (Mantel correlation, r=0.313, *P*=0.001; figure 2C) and by the abundance of non-*Helicobacter Proteobacteria* (Mantel correlation, r=0.447, *P*=0.001; figure 2D).

Regarding *Helicobacter* spp. in chronic gastritis, the mean relative abundance of this genus was 41.7%, but varied considerably between patients from 0.01% to 94.9% (figure 2A). The relative abundance of *Helicobacter* was inversely correlated with the abundance of non-*Helicobacter Proteobacteria* (r=−0.59, *P*<0.0001), *Firmicutes* (r=−0.49, *P*<0.0001), *Bacteroidetes* (r=−0.43, *P*<0.0001) and *Actinobacteria* (r=−0.54, *P*<0.0001; online supplementary table S5). In contrast, the great majority of patients with gastric carcinoma (80%) had a relative abundance of *Helicobacter* below 5%, including eight patients in which *Helicobacter* reads were not detected by NGS. The abundance of *Helicobacter* in gastric carcinoma was correlated with that of *Bacteroidetes* and *Fusobacteria* (see online supplementary table S5).

Overall, these results show that for high taxonomic levels the stomach microbial communities differ in chronic gastritis and gastric carcinoma, suggesting that major changes also occur at lower taxonomic levels. Additionally, our data validate that *Helicobacter* exists in the gastric carcinoma microbiota as a low abundant or absent genus.

### Specific microbial taxa are associated with gastric carcinoma

**Figure 3:**
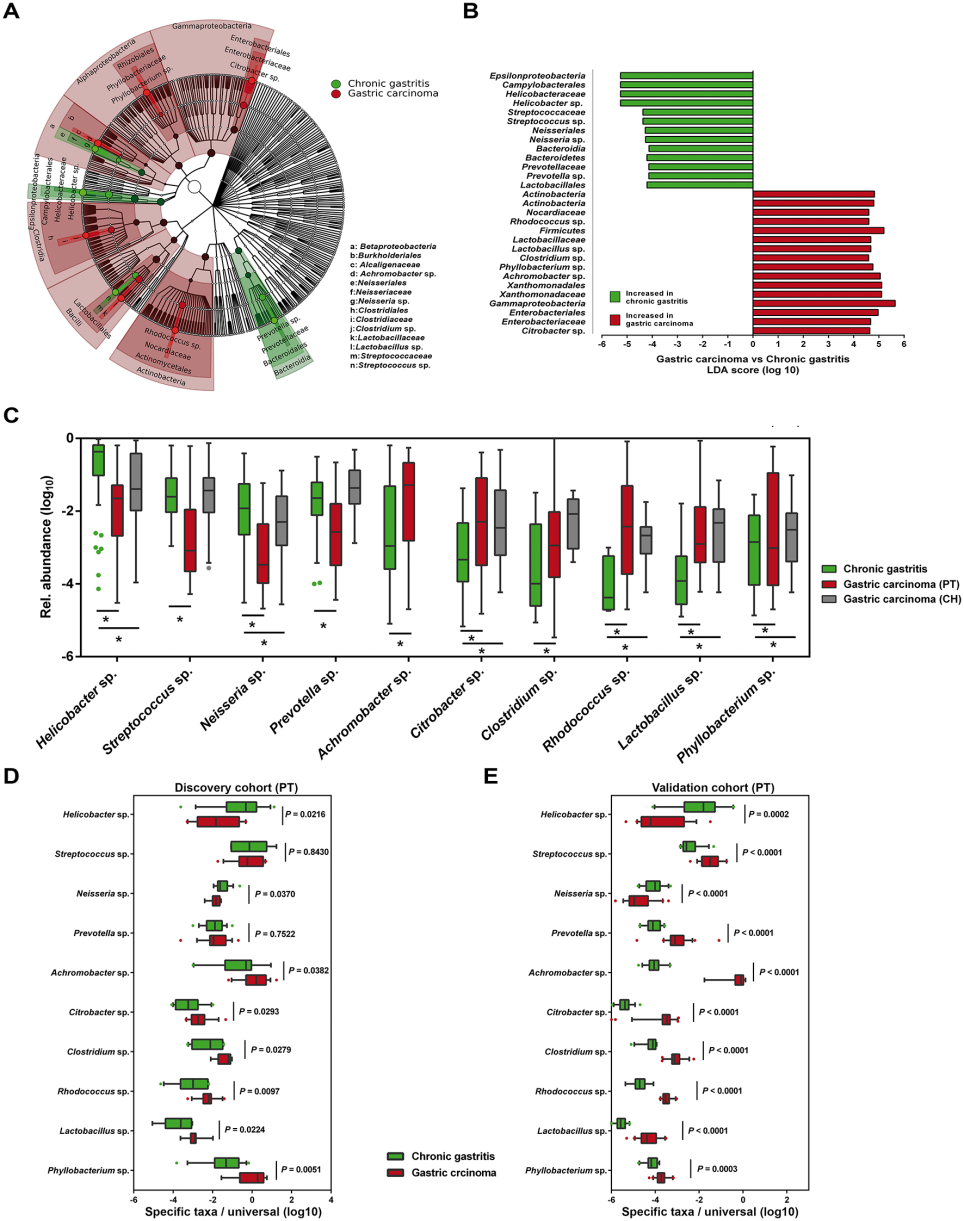
Microbial taxa associated with gastric carcinoma. (A) Cladogram representation of the gastric microbiota taxa associated with chronic gastritis and gastric carcinoma. (B) Association of specific microbiota taxa with the group of chronic gastritis and gastric carcinoma by linear discriminant analysis (LDA) effect size (LEfSe). Green indicates taxa enriched in chronic gastritis group and red indicates taxa enriched in gastric carcinoma group. (C) Relative abundance of the 10 genera differentially enriched in the two clinical settings across Portuguese discovery and Chinese validation cohorts. *Significance obtained by LEfSe analysis at *P*<0.05. (D,E) Validation of LEfSe results by quantitative PCR (qPCR) of the 10 genera differentially enriched in the discovery cohort (D) and in the Portuguese validation cohort (E). Significance was obtained by Student’s t-test.

To identify the most relevant taxa responsible for the differences between clinical diagnoses, we conducted LEfSe analysis.^27^ This analysis identified 29 taxa, including 10 genera, which were differentially abundant in the two patient groups (figure 3A,B). In gastric carcinoma, an enrichment in *Proteobacteria* taxa was observed, including the genera *Phyllobacterium* and *Achromobacter* and the families *Xanthomonadaceae* and *Enterobacteriaceae*. Although no specific genus could be identified within the *Xanthomonadaceae*, in the *Enterobacteriaceae*, the genus *Citrobacter* was identified as being significantly enriched in gastric carcinoma. Additionally, *Lactobacillus*, *Clostridium* and *Rhodococcus* were also significantly more abundant in gastric carcinoma. *Helicobacter*, *Neisseria*, *Prevotella* and *Streptococcus* were most abundant in the microbiota of patients with chronic gastritis. Results of the LEfSe analysis in the age-matched subset closely recapitulated the bacteria taxa differentially abundant in the two patient groups (see online supplementary figure S4F).

To show that relationships among disease-associated taxa did not depend on differences observed in the abundance of *Helicobacter,* we conducted a reanalysis subtracting the *Helicobacter* reads from the data set. Considering the same parameters in the LEfSe analysis, we confirmed the enrichment of *Streptococcus, Prevotella* and *Neisseria* in chronic gastritis (see online supplementary figure S7). Additionally, we identified an enrichment in two *Proteobacteria* taxa, *Novosphingobium* and *Pasteurellales*, and in two *Bacteroidetes* families, *Chitinophagaceae* and *Saprospirae*. In gastric carcinoma, no additional taxa were detected after removing the *Helicobacter* reads from the data set.

To validate gastric carcinoma-enriched and depleted taxa, we used NGS data from an independent Chinese cohort of 79 gastric carcinoma cases. In this data set, and in agreement with the results obtained in the Portuguese discovery cohort, we could detect statistically significant enrichment in *Citrobacter*, *Rhodococcus*, *Lactobacillus* and *Phyllobacterium*, and depletion in *Helicobacter* and *Neisseria* (figure 3C). *Clostridium* reads were enriched in gastric carcinoma cases from the Chinese population, although not reaching statistical significance in the LEfSe analysis. *Achromobacter* reads were not detected in the Chinese validation cohort.

To demonstrate that our data were not biased by the microbiota profiling pipeline used, LEfSe results were validated by qPCR in the Portuguese discovery cohort using both genus-specific and universal primers. We confirmed significant decreases in the abundance of *Helicobacter* and *Neisseria*, and significant increases of *Achromobacter*, *Citrobacter*, *Phyllobacterium*, *Clostridium*, *Rhodococcus* and *Lactobacillus* in gastric carcinoma in comparison with chronic gastritis (figure 3D). We have additionally used a second validation cohort from Portugal, and with the exception of *Prevotella* and *Streptococcus*, we were able to confirm the alterations in the abundance of the eight genera as identified by the original LEfSe analysis (figure 3E).

Next, we compared gastric carcinoma cases and chronic gastritis control subjects for the prevalence of specific taxa. As shown in table 1, the six genera significantly enriched in gastric carcinoma and identified by LEfSe analysis were also significantly more prevalent in patients with gastric carcinoma than in patients with chronic gastritis. In logistic regression models with carriage of the genera *Phyllobacterium*, *Achromobacter, Citrobacter, Lactobacillus, Clostridium* or *Rhodococcus* as the independent variables, and gastric carcinoma as the dependent variable, the ORs for gastric carcinoma were 3.5 (95% CI 1.7 to 7), 20.5 (95% CI 7.4 to 59), 9.9 (95% CI 4.3 to 23), 6.3 (95% CI 2.9 to 14), 5.7 (95% CI 2.2 to 15) and 4.2 (95% CI 1.7 to 11), respectively. The associations remained significant after adjustment for age and sex.

**Table 1:** Relative abundance and prevalence of selected microbial taxa in patients with chronic gastritis and gastric carcinoma in the discovery cohort

Taxa (phylum; class; order; family; genus)	Relative abundance (%)*			Prevalence (%)†			Univariate OR (95% CI)	Multivariate‡ OR (95% CI)
	Chronic gastritis	Gastric carcinoma	P*§*	Chronic gastritis	Gastric carcinoma	P¶		
*Proteobacteria; Alphaproteobacteria; Rhizobiales; Phyllobacteriaceae;* *Phyllobacterium* sp.	0.2	5.4	<0.0001	34.6	64.8	0.001	3.5 (1.7 to 7)	3.7 (1.2 to 11)
*Proteobacteria; Betaproteobacteria; Burkholderiales; Alcaligenaceae;* *Achromobacter* sp.	2.1	11.1	<0.0001	24.7	87.0	<0.0001	20.5 (7.4 to 59)	56.3 (9.5 to 333)
*Proteobacteria; Betaproteobacteria; Neisseriales; Neisseriaceae;* *Neisseria* sp.	3.9	0.4	<0.0001	92.6	75.9	<0.0001	0.25 (0.09 to 0.7)	0.27 (0.1 to 1)
*Proteobacteria; Epsilonproteobacteria; Campylobacterales; Helicobacteraceae;* *Helicobacter* sp.	41.7	5.9	<0.0001	100	85.2	<0.0001	0.0 (0.0 to 0.4)	NA
*Proteobacteria; Gammaproteobacteria; Enterobacteriales; Enterobacteriaceae; Citrobacter* sp.	0.2	4.3	<0.0001	30.9	81.5	<0.0001	9.9 (4.3 to 23)	6.5 (2.1 to 20)
*Firmicutes; Bacilli; Lactobacillales; Lactobacillaceae;* *Lactobacillus* sp.	0.025	4.7	<0.0001	19.7	61.1	<0.0001	6.3 (2.9 to 14)	3.1 (1.0 to 10)
*Firmicutes; Bacilli; Lactobacillales; Streptococcaceae; Streptococcus* sp.	7.7	2.9	<0.0001	97.5	92.6	0.217	NA	NA
*Firmicutes; Clostridia; Clostridiales; Clostridiaceae;* *Clostridium* sp.	0.046	3.7	<0.0001	8.6	35.2	<0.0001	5.7 (2.2 to 15)	7.8 (2.0 to 31)
*Actinobacteria; Actinobacteria; Actinomycetales; Nocardiaceae;* *Rhodococcus* sp.	0.002	3.3	<0.0001	9.9	31.5	0.003	4.2 (1.7 to 11)	3.8 (1.0 to 15)
*Bacteroidetes; Bacteroidia; Bacteroidales; Prevotellaceae;* *Prevotella* sp.	5.0	2.0	<0.0001	97.5	90.7	0.104	NA	NA

*Average relative abundance of specific taxa in subjects containing that taxa.

†Percentage of subjects carrying the specific taxa.

‡Multivariate logistic regression analysis with non-carriers as reference adjusted for age and gender.

§P values obtained through linear discriminatory analysis effect size (LEfSe).

¶P values obtained by Fisher’s exact test.

NA, not assessed.

### Microbial dysbiosis is associated with gastric carcinoma

**Figure 4:**
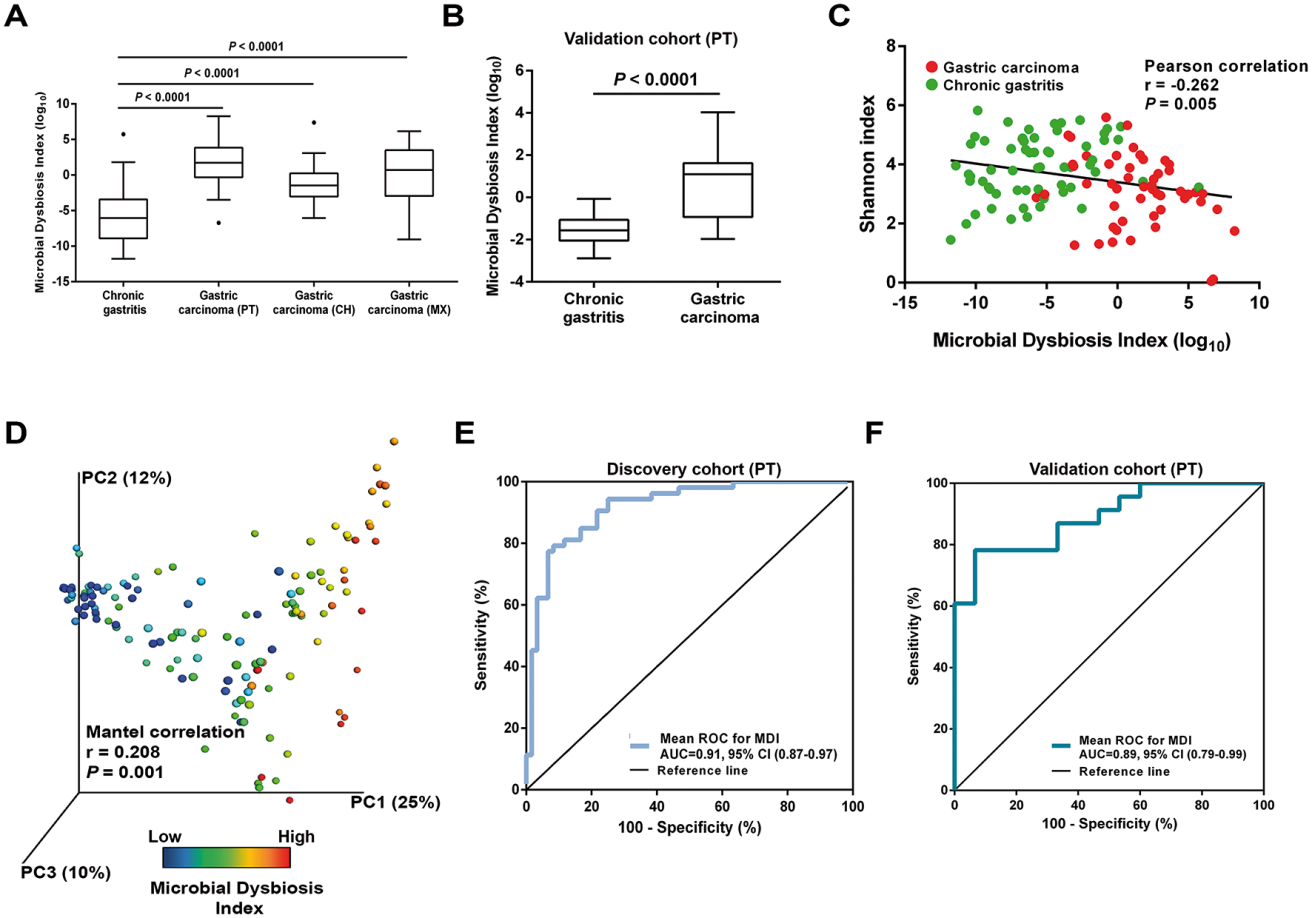
Microbial dysbiosis is associated with gastric carcinoma. (A) Box plot showing the MDI in the discovery cohort and in the Chinese and Mexican validation cohorts. Significance was obtained by one-way analysis of variance (ANOVA) corrected with Holm-Sidak test for multiple comparisons. (B) Box plot showing the MDI of the Portuguese validation cohort. Significance was obtained by Student’s t-test. (C) Negative Pearson’s correlation between MDI and Shannon index. (D) Principal coordinate analysis (PCoA) plot of the weighted UniFrac distance coloured by increasing MDI. The percentage of diversity captured by each coordinate is shown. Mantel correlations controlled with 10^4^ permutations were used to compare distances. (E,F) ROC curves analysis to evaluate the discriminatory potential of MDI in gastric carcinoma detection in the discovery cohort (E) and in the Portuguese validation cohort (F). AUC, area under the curve; MDI, microbial dysbiosis index; ROC, receiver operating characteristic.

We next combined the 10 most relevant taxa that characterised each group of patients and calculated the microbial dysbiosis index (MDI).^31^ The gastric microbiota of patients with gastric carcinoma had a higher MDI than that of patients with chronic gastritis both in the discovery cohort and in the validation cohorts (*P*<0.0001; figure 4A). Similar findings were observed in the age-matched subset of the discovery cohort (see online supplementary figure S4G). Likewise, significantly higher MDI was observed in the microbiota of patients with gastric carcinoma in comparison with that of patients with chronic gastritis, as assessed using qPCR in the Portuguese validation cohort (*P*<0.0001; figure 4B). The MDI showed an inverse correlation with the alpha diversity (r=−0.262, *P*=0.005; figure 4C) and a direct correlation with the beta diversity (r=0.208, *P*=0.001; figure 4D), resulting in a clear differentiation gradient among samples. These results demonstrate that the gastric carcinoma microbiota has a high degree of dysbiosis, consistent with reduced bacterial diversity.

We also evaluated whether the MDI could be used to discriminate between chronic gastritis and gastric carcinoma. In receiver operating characteristics (ROC) analysis, the MDI showed excellent performance in identifying gastric carcinoma, yielding an area under the curve (AUC) of 0.91 and 0.89 for the Portuguese discovery and validation cohorts, respectively (figure 4E,F). The MDI exhibited improved sensitivity and specificity to detect gastric carcinoma when compared with the use of single taxa (see online supplementary figure S8).

Since in the validation cohorts we could not confirm the differential abundance of *Prevotella* and *Streptococcus*, we recalculated the MDI excluding these genera. This analysis confirmed higher levels of dysbiosis in the gastric carcinoma microbiota in all cohorts, and similar AUCs in the ROC analysis (see online supplementary figure S9).

### The gastric carcinoma microbiota is characterised by nitrosating bacteria

To infer the metagenome functional content based on the microbial community profiles obtained from the 16S rRNA gene sequences we used PICRUSt.^28^ Overall, the microbial communities present in patients with gastric carcinoma and chronic gastritis could be distinguished based on their functions (see online supplementary figure S10A). The predicted KEGG pathways significantly enriched in gastric carcinoma included membrane transport, carbohydrate metabolism, transcription, xenobiotics biodegradation and metabolism, cellular processes and signalling, metabolism, signal transduction, amino acid metabolism and lipid metabolism (see online supplementary table S6).

**Figure 5:**
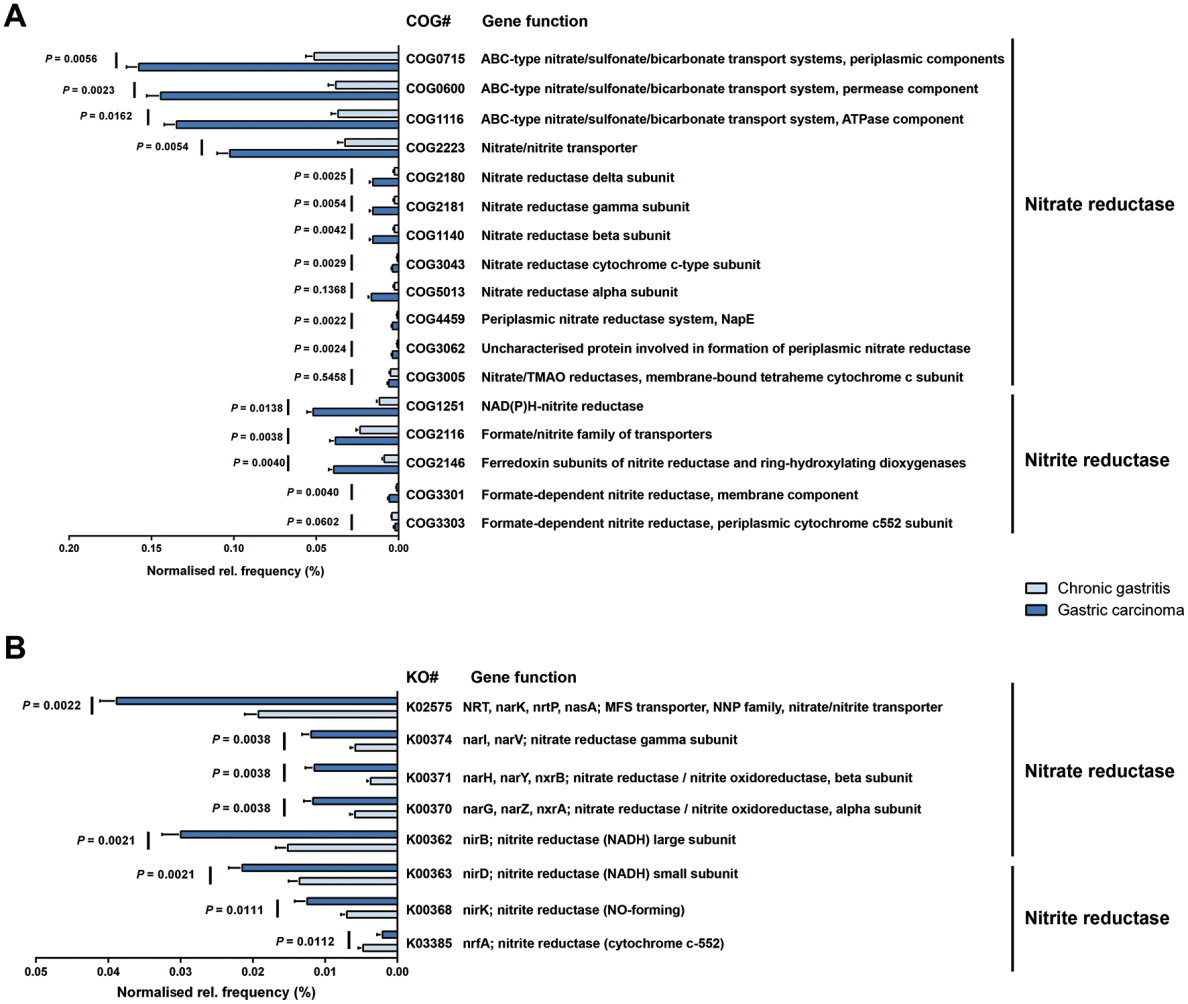
The gastric carcinoma microbiota is characterised by nitrosating bacteria. Functional classification of the predicted metagenome content of the microbiota of chronic gastritis and gastric carcinoma using (A) COG and (B) KO. The normalised relative frequency of nitrate reductase and nitrite reductase in patients with chronic gastritis and gastric carcinoma is shown. Significance was considered for adjusted *P*<0.05. COG, Clusters of Orthologous Groups; KO, Kyoto Encyclopedia of Genes and Genome (KEGG) orthology; NADH, nicotinamide adenine dinucleotide; NO, nitric oxide; TMAO, trimethylamine N-oxide.

Because it has been hypothesised that nitrate-reducing bacterial species contribute to gastric malignant transformation by increasing intragastric concentrations of nitrite and *N*-nitroso compounds, we next compared chronic gastritis and gastric carcinoma regarding the microbial functional features involved in those metabolic reactions (see online supplementary table S7). The full reconstitution of the metagenomes showed that the functional composition of the total gastric carcinoma microbiota had increased nitrate reductase functions, which promote the reduction of nitrate to nitrite, and nitrite reductase functions, which promote the reduction of nitrite to nitric oxide, when compared with that of the chronic gastritis (figure 5A,B). Similar results were obtained when the 10 genera differentially abundant in the two patient groups were analysed (see online supplementary figure S10B,E). Collectively, these data provide evidence that a microbial community with genotoxic potential is present in gastric carcinoma.

## Discussion

We have profiled the gastric microbiota associated with chronic gastritis and gastric carcinoma in the largest and most in-depth study to date. We have demonstrated that the gastric microbiota composition in patients with gastric carcinoma is significantly different from that of patients with chronic gastritis. Gastric carcinoma dysbiosis was consistent with a microbial community with genotoxic potential, characterised by reduced microbial diversity, reduced *Helicobacter* abundance and over-representation of new bacterial genera. The major findings revealed in the Portuguese discovery cohort were confirmed in additional validation cohorts from multiple geographic locations.

In our study, the gastric microbial communities in gastritis and carcinoma were structurally different, with decreased alpha diversity in carcinoma. Our findings are supported by previous data pointing to lower bacterial diversity among five patients with gastric cancer compared with five patients with non-atrophic gastritis.^15^ Also supporting our data, and while our paper was in revision, another paper was published in *Gut* that identified significant decreases in microbial richness in intestinal metaplasia and in gastric carcinoma compared with superficial gastritis.^32^ Reduced microbial diversity has now been recognised as a feature of disease states, including inflammatory diseases and cancer.^31 33 34^ For example, patients with colorectal cancer had decreased overall microbial community diversity in comparison to healthy controls.^34^

In terms of the composition of the gastric microbiota, *Proteobacteria*, *Firmicutes*, *Bacteroidetes*, *Actinobacteria* and *Fusobacteria* were the five dominant phyla in the stomach, in accordance with previous descriptions.^12 14 20^ At the phylum level, we have already identified differences between the two patient groups, with increased abundance of non-*Helicobacter Proteobacteria*, *Firmicutes* and *Actinobacteria* in cancer specimens. Importantly, by applying the LEfSe algorithm that was validated for high-dimensional microbiome data sets, we were able to determine the bacterial taxa that most likely explain differences between clinical diagnoses.^27^ Additionally, in this study, the major taxonomic differences that were detected after analyses of sequencing-generated and bioinformatics-treated data were further validated by real-time qPCR assays.

In chronic gastritis, and as expected, *Helicobacter* was detected as the most abundant genus. *Streptococcus, Prevotella* and *Neisseria* were also found significantly overabundant in this patient group, although *Streptococcus* and *Prevotella* could not be confirmed by qPCR. Nevertheless, these genera have been identified earlier in *H. pylori*-positive and negative gastritis by 16S rDNA and rRNA sequencing, and by culture from gastric juice and gastric biopsies.^12 14 35 36^ In fact, they are among the five most commonly found genera in the non-neoplastic stomach.^12 20 37^ *Streptococcus*, *Prevotella* and *Neisseria* are commensals of the oral cavity and oesophagus and whether they constitute transient or active resident stomach microbes is not yet clarified. Interestingly, in a study that compared the gastric microbiota compositions in *H. pylori*-positive individuals from two populations with high and low gastric cancer risks in Colombia, *Neisseria* and *Streptococcus* were among the genera that occurred more abundantly in individuals from the low gastric cancer risk region.^38^

In gastric carcinoma, there was a significant decrease in *Helicobacter* abundance, and several taxa were found to be significantly more abundant. These included *Citrobacter*, *Clostridium*, *Lactobacillus*, *Achromobacter* and *Rhodococcus*, which reside in the intestinal mucosa as commensals but can be opportunistic pathogens.^39 40^ *Phyllobacterium*, which are environmental bacteria commonly found in plant roots, were too identified at higher abundance in gastric carcinoma.^41^ All genera significantly overabundant in gastric carcinoma were also significantly more prevalent in gastric carcinoma cases than in chronic gastritis control patients, and these associations remained significant after adjustment for age and sex. In line with our results, in a study that combined terminal restriction fragment length polymorphism with 16S rRNA gene cloning and sequencing, *Lactobacillus* was one of the dominating genera in 10 Swedish patients with gastric cancer.^16^ Additionally, the use of the microarray G3 PhyloChip to characterise the stomach microbiota of Mexican patients revealed a trend towards the increase of a *Lactobacillus* sp from non-atrophic gastritis, to intestinal metaplasia, to gastric cancer.^15^ Moreover, *Citrobacter*, *Clostridium* and *Lactobacillus* have all been cultured from the gastric juice of achlorhydric patients, patients undergoing acid suppression therapy and patients with gastric cancer.^42–44^ Interestingly, infection with *Citrobacter rodentium* species increases epithelial cell proliferation and promotes colonic tumour formation in genetically susceptible mice as well as in chemically initiated colon carcinogenesis.^45 46^

The integration of data from the most relevant genera that characterised each patient group allowed us to calculate the dysbiosis index that showed excellent capacity to discriminate between gastritis and gastric carcinoma. Furthermore, the dysbiosis index had improved sensitivity and specificity to detect gastric carcinoma in comparison with the use of single genera, which suggests that changes in the microbial community rather than individual taxa contribute to gastric carcinoma development.

After having analysed the diversity and composition of the gastric microbiota and the microbial features associated with gastric carcinoma, we addressed the functional features of the microbiota. Specifically, we demonstrated that in comparison with chronic gastritis, the gastric carcinoma microbiota has increased nitrate reductase and nitrite reductase functions. This observation is compatible with the hypothesis that during carcinogenesis, changes in the stomach mucosa that lead to decreased acid secretion allow the growth of bacteria that are able to reduce nitrate to nitrite, a precursor of carcinogenic *N*-nitroso compounds.^2^

Taken together our results and previously published data, we propose that colonisation with bacteria other than *H. pylori*, namely gut commensals, contributes to alter the equilibrium between the ‘resident’ gastric microbiota and the host. This dysbiotic microbial community, by sustaining the gastric inflammatory process, and through its intrinsic genotoxic potential, may augment the risk for *H. pylori*-related gastric carcinoma development. In line with our proposal, experimental evidence in the INS-GAS model showed that commensal intestinal bacteria play a role in the promotion of gastric cancer.^12 13^ Lertpiriyapong *et al* showed that mice harbouring a complex intestinal microbiota, and mice colonised with a restricted intestinal microbiota (that includes *Clostridium* and *Lactobacillus*), had an accelerated onset and progression of gastric cancer secondary to *H. pylori* infection. These mice also developed more severe gastric histopathology and higher expression levels of proinflammatory genes in comparison to germ-free mice (infected or not with *H. pylori*) and mice harbouring a complex or a restricted intestinal microbiota.^11^

Although our study is limited by its retrospective nature, and by the low number of patients with true premalignant lesions, our findings are consistent with a shift in the gastric microbial community structure along gastric carcinogenesis. In this sense, prospective follow-up studies of patients with premalignant lesions, successfully eradicated or not for *H. pylori* infection, would be crucial to ascertain the pathogenic effect of microbial dysbiosis in the progression to carcinoma. Additional studies to address the effect of dysbiosis or of candidate bacterial species in an animal model of gastric carcinogenesis can also be considered, and in that regard, a humanised mouse model that better mimics the human immune response could be particularly informative. Ultimately, understanding the microbiota dynamics along gastric carcinogenesis may impact gastric carcinoma prevention and treatment strategies of patients with precancerous disease.
