# Supplementary material for: Gastric microbial community profiling reveals a dysbiotic cancer-associated microbiota
Source: Gut. 2017 Nov 4;67(2):226–36. doi: 10.1136/gutjnl-2017-314205 (PMC5868293; doi:10.1136/gutjnl-2017-314205)
Supplement: Supplementary file 1 [file gutjnl-2017-314205supp001.pdf]

Gastric microbial community profiling reveals a dysbiotic cancer-associated microbiota

Rui M. Ferreira[1,2], Joana Pereira-Marques[1,2,3], Ines Pinto-Ribeiro[1,2,4], Jose L. Costa[1,2,4], Fatima Carneiro[1,2,4,5], Jose C. Machado[1,2,4], Ceu Figueiredo[1,2,4*]

[1]i3S - Instituto de Investigação e Inovação em Saúde, Universidade do Porto, Porto, Portugal; [2]Ipatimup - Institute of Molecular Pathology and Immunology of the University of Porto, Porto, Portugal; [3]ICBAS - Institute of Biomedical Sciences Abel Salazar, University of Porto, Portugal; [4]Faculty of Medicine of the University of Porto, Porto, Portugal; and [5]Department of Pathology, Centro Hospitalar São João, Porto, Portugal

SUPPLEMENTARY MATERIAL

SUPPLEMENTARY MATERIALS AND METHODS

Helicobacter pylori detection

DNA extracted from gastric tissue was used for *H. pylori* detection by amplification of the *vacA* and *cagA* genes, using biotin-labeled primers as previously described.[1] The detection of amplicons was performed by reverse hybridization onto a line probe assay (Labo Bio-Medical Products, Rijswijk, The Netherlands). DNA of *H. pylori* strains with known genotypes were used as templates for positive and negative controls.

16S rRNA gene sequencing

The PCR reactions were performed in 25µL containing 1X AmpliTaq Gold 360 Master Mix (Applied Biosystems, Foster City, CA) and 0.4µM of forward and reverse primers. PCR was performed with 9 min of predenaturation at 95ºC, followed by 25 cycles of 30 seconds at 95ºC, 45 seconds at 52ºC, and 45 seconds at 72ºC. Final extension was performed for 10 minutes at 72ºC. Amplicons of approximately 280 bp were visualized and purified using the E-Gel SizeSelect Agarose Gels (Life Technologies, Foster City, CA), their concentration was determined with Qubit dsDNA HS Assay Kit (Life Technologies) and the respective size distribution with Qiaxcel DNA screening (Qiagen, Germany). Equal concentrations were used for library preparation to incorporate adaptor and barcode sequences. Next-generation sequencing was performed in the Ion PGM Torrent platform using Ion 316v2 Chips (Life, Technologies), following manufacturer’s protocols. Briefly, 50ng of amplified DNA was used to ligate the Ion Torrent A (containing the sequencing primer ligation site) and P1 adaptors (the site for Ion Sphere Particles-ISP ligation). Afterwards, the DNA molecules entered in a process of clonal amplification through an emulsion PCR (emPCR) using the OneTouch2 instrument (Ion Torrent, Life Technilogies). During emPCR single DNA fragments become bound via the specific adaptor to single ISPs. This process leads to coating of each ISP with millions of copies of a single DNA fragment. To ensure only coated ISPs proceed for sequencing, an enrichment step was performed using the OneTouch ES instrument (Ion Torrent, Life Technologies). PCR negative controls containing Microbial DNA-free water

(Qiagen), instead of DNA were processed as above mentioned. Data were deposited in Sequence Read Archive (PRJNA413125).

Target re-sequencing quality control

The number of spurious OTUs and the number of biological meaningful OTUs were obtained according to similarity shared with sequences of the Greengenes Named Isolated database (Supplementary Figure S2).[2] OTUs were classified as “Named” (≥ 97% match from the reference database), “Chimeric” (<97% match to the reference database and chimeric with high confidence) and “Missing” (<97% match with low confidence or a biological sequence missing from the database). Consistency in the values determined for alpha-diversity, beta-diversity and relative abundances of *Proteobacteria*, *Firmicutes*, *Bacteroidetes* and *Fusobacteria* across amplification and sequencing sets were evaluated by intraclass correlation coefficient in 32 samples derived from 12 chronic gastritis and 4 gastric carcinoma subjects (Supplementary Table S3).

NGS data analysis of the Chinese and Mexican validation cohorts

The gastric carcinoma raw paired-end reads of the 16S rRNA gene of the V3-V4 region were downloaded in FASTQ format from Sequence Read Archive (SRA) from the study SRP080738 (BioProject PRJNA310127). In total 80 samples from China and 54 samples from Mexico were downloaded. Reads were analysed using the UPARSE pipeline, as described for the discovery cohort. Chimeric reads were reference-removed using Uchime. Each OTU was taxonomically assigned using Uclust, considering a minimum of 90% of similarity to a reference database (Greengenes Named Isolate database, release August 2013). After quality filtering, OTU construction, chimera filtering, and removal of non-bacteria sequences, two samples (one from each cohort) were removed due to low number of reads (< 1000 sequences). The final set comprised 79 gastric cancer samples from China and 53 from Mexico (Supplementary Table S2). Noteworthy, the number of reads that could not be assigned to bacteria in these datasets were remarkably high (47.8% and 38.2% for China and Mexico, respectively) in comparison to those observed in the Portuguese discovery cohort (0.38%).

Real-time quantitative PCR (qPCR)

qPCR assays were performed using PowerUp SYBR Green Master Mix (Applied Biosystems) using different sets of primers. Two different assays were used for quantification, a universal assay (composed by universal primers targeting a conserved region of the 16S rRNA gene) and a specific assay (composed by genus-specific primers targeting the 16S rRNA gene) (Supplementary Table S2). The assays were designed to obtain the highest degree of specificity by comparison with sequences of Greengenes Named Isolate (release August 2013).

qPCR mixtures were prepared to a final volume of 10 µL, containing 1x PowerUp SYBR Green Master Mix, 1 µM of forward and reverse primers (Invitrogen, Foster City, CA), 2 µL of Microbial DNA-Free Water (Qiagen) and 1 µL of DNA. The qPCR was performed in a 7500 Fast Real-Time PCR System (Applied Biosystems) with the following conditions: 2 minutes at 50ºC, 10 minutes at 95ºC, followed by 40 cycles of denaturation at 95ºC for 15 seconds and annealing/extension at 60ºC for 1 minute. The amplification steps were followed by a melt dissociation step to check for nonspecific product formation. This step comprises an additional cycle of 95ºC for 15 seconds, 60ºC for 1 minute, 95ºC for 30 seconds and 60ºC for 15 seconds. In addition, the PCR product purity was also controlled by Agarose gel electrophoresis. Two replicates were performed for each sample. To exclude any potential environmental contaminant in PCR reactions, blanks were prepared using Microbial DNA-Free Water (Qiagen) instead of DNA.

The relative standard curve method was used to quantify the specific genera in patients with chronic gastritis and with gastric carcinoma. To create standard curves, amplicons of each designed assay were cloned into pGEM-T easy vector system (Promega, Madison, WI). Dilution series of known plasmid concentrations were used to create a standard curve for each assay by plotting the log of each known concentration in the dilution series against the determined C_T (threshold cycle) value.

From the standard curves, the reaction parameters (slope, *y*-intercept, correlation coefficient and efficiency) were obtained and the concentration of the target bacteria species was extrapolated. For each assay, the PCR efficiencies (determined as $10^{(-1/slope)}$ - 1) obtained were: Universal – 91%; *Helicobacter* sp. – 100%; *Streptococcus* sp. – 115%; *Neisseria* sp. – 92%; *Prevotella* sp. – 101%; *Achromobacter* sp. – 114%; *Citrobacter* sp. – 97%; *Clostridium* sp. – 83%; *Rhodococcus*

sp. – 102%; *Lactobacillus* sp. – 91%; and *Phyllobacterium* sp. – 100%. The abundance of each genus was determined by the log10 ratio between the DNA concentration determined for the specific assay and the DNA concentration determined for the universal assay. The Student's t-test was used to compare the abundance of genus between chronic gastritis and gastric carcinoma cases.

Interpolation of linear and non-linear models and correlations

Interpolation of non-linear models was used to explain the relationship between two variables. These models were determined using the following equation (second order polynomial):

$$y = B0 + B1x + B2x^2$$

where x represents the relative abundance of *Helicobacter* spp. and y represents the Shannon index. B0, B1 and B2 represent the polynomial coefficients. Non-linear models were shown with the 95% confidence intervals lines. Deviation of data from non-linear models was evaluated by performing run tests, which apply a series of consecutive points that are above or below the interpolation curve and determine the probability that data diverge significantly (at a P- value ≤ 0.05).

The normality of the data was evaluated using the Kolmogorov-Smirnov test. Correlations between variables were performed using Pearson's correlation (for normally distributed data) or Spearman's rank correlation (for non-normally distributed data).

Logistic regression and receiver operating characteristic (ROC) analyses

The risk of gastric carcinoma was evaluated by multivariate logistic regression analysis, using clinical outcome as dependent variable and taxa prevalence as independent variable, adjusted for age and gender. The Hosmer-Lemeshow test was used to control goodness-of-fit of logistic regression models using 8 degrees of freedom and 10 steps. ROC curves were constructed to evaluate the ability of relative abundance of genera and of MDI to detect gastric carcinoma. The relative abundance was defined as the raw counts of genus-level taxa detected in at least one sample that were then normalized per sample by the total counts of all taxa in that sample so that the resulting relative abundances sum 100%. A mean ROC curve was reported including the 95%

confident intervals. The best discrimination was determined by using the highest area under the curve at a significance value of $P \leq 0.05$.

Microbial dysbiosis index (MDI)

The MDI was determined as the log transformation of the ratio between the total abundance of genera increased in gastric carcinoma and the total abundance of genera decreased in gastric carcinoma.[3] Unless otherwise stated, *Rhodococcus* spp., *Lactobacillus* spp., *Clostridium* spp., *Phyllobacterium* spp., *Achromobacter* spp. and *Citrobacter* spp. were included as increased in gastric carcinoma, and *Helicobacter* spp., *Neisseria* spp., *Prevotella* spp. and *Streptococcus* spp. were included as decreased in gastric carcinoma.

Functional metagenome predictions

To predict the functional metagenome, we captured OTU representative sequences from Greengenes database using USEARCH global alignment command. Thus, only sequences with best hit to the corresponding taxonomy were kept, i.e. all OTUs with a hit of 97% similarity to the reference database were preserved. The reconstruction of the functional metagenome content was performed using PICRUSt.[4] This pipeline is a computational method to predict the gene function composition of a metagenome using 16S rRNA sequencing data and a database of reference genomes. The abundance of each OTU was corrected to reflect the true bacterial abundance by normalizing the 16S rRNA copy number for each OTU. The accuracy of the predicted metagenomes was determined by the NSTI (nearest sequenced taxon index). NSTI quantifies the availability of nearby representative genomes for each microbiome sample and is determined as the sum of phylogenetic distances for each microbe in the OTU table to its adjacent relative with a sequenced reference genome. Low NSTI values (closer to zero) indicate better accuracy in metagenome prediction.[4] Predicted functional genes were classified into clusters of orthologous groups (COG) that contains prokaryotic proteins of complete genomes or into Kyoto encyclopedia of genes and genomes orthology (KO) and submitted to a two-group comparison (gastritis vs carcinoma) using STAMP.[5] Differences in COG and KO relative frequencies were determined by White's non-parametric *t* test[6] with a Benjamini-Hochberg false discovery rate correction to adjust *P*-values for

multiple testing. The gene functions classified by KO were further categorized into KEGG Pathways using PICRUSt. The enrichment of predicted KEGG Pathways was assessed by White's non-parametric t test with a Benjamini-Hochberg false discovery rate correction and by LEfSe analysis. The functional contribution of taxa to selected predicted functions was evaluated with metagenome_contributions.py, a built-in function in PICRUSt.

SUPPLEMENTARY FIGURES

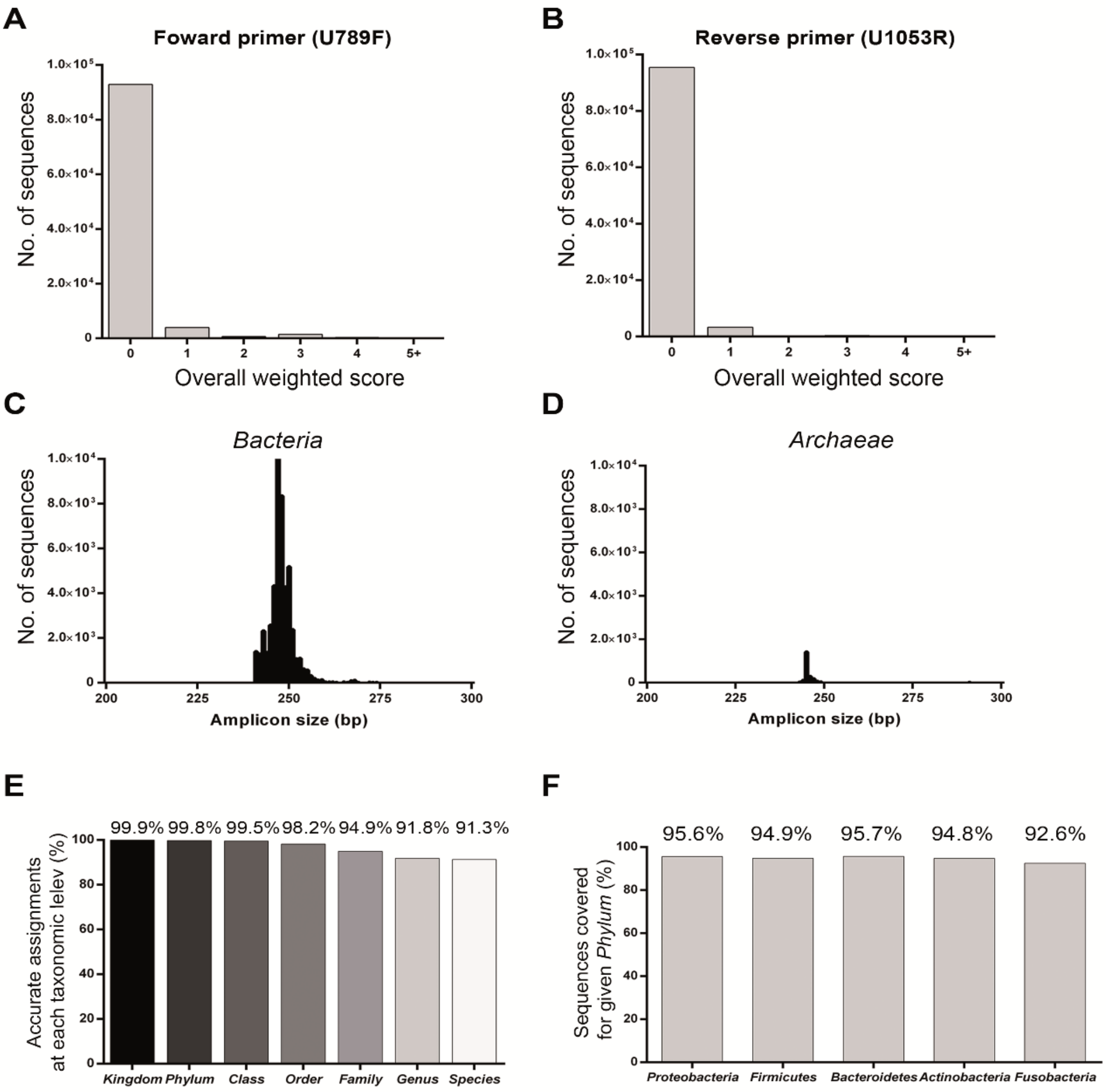

Supplementary Figure S1. Performance of the selected primers (U789F and U1053R) for 16S rRNA V5-V6 next-generation sequencing evaluated with Primer Analysis Pipeline implemented in PrimerProspector software package.[7] Number of sequences covered by the **(A)** forward and by the **(B)** reverse primer. The overall weighted score represents the performance of the primers by measuring penalties of primers to match reference sequences. Overall weighted score = non-3' mismatch × 0.40 + 3' mismatch ×1 + non-3' gaps × 1 + 3' gaps × 3. Simulated reads using the U789F and U1053R showed high number of **(C)** *Bacteria* sequences and low number of **(D)** *Archaea* sequences. Taxonomic coverage of simulated reads by **(E)** taxonomic level and by most relevant **(F)** *Phylum* represented in the gastric microbiota. The representative OUT sequences (clustered at 97% similarity) of the Greengenes Named Isolate database was used as reference for this analysis.

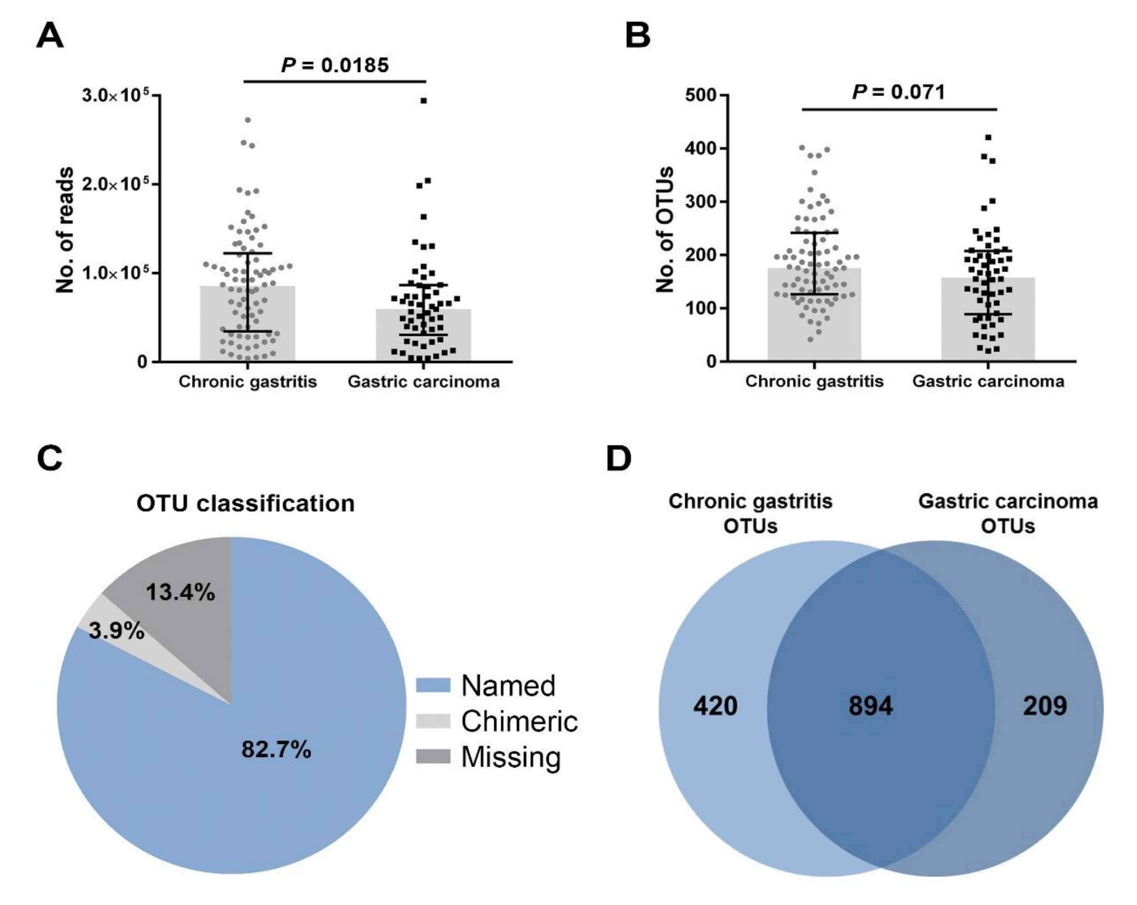

Supplementary Figure S2. Performance of the UPARSE pipeline[6] in 16S rRNA V5-V6 reads derived from 81 chronic gastritis and 54 gastric carcinoma patients. Number of reads **(A)** and OTUs **(B)** after quality filtering. Mann-Whitney test was used to compare chronic gastritis and gastric carcinoma patients in respect to the number of reads or OTUs. Data is shown as median with interquartile range. **(C)** Pie chart showing the average fraction of OTUs classified accordingly to similarity shared with sequences of the Greengenes Named Isolate database, as previously reported[6, 14]: Named (≥ 97% match from the reference database), Chimeric (< 97% match to the reference database and chimeric with high confidence) and Missing (< 97% match with low confidence or a biological sequence missing from the database). **(D)** Venn diagram showing the number of OTUs exclusively identified in each group of patients and OTUs shared by the two groups of patients.

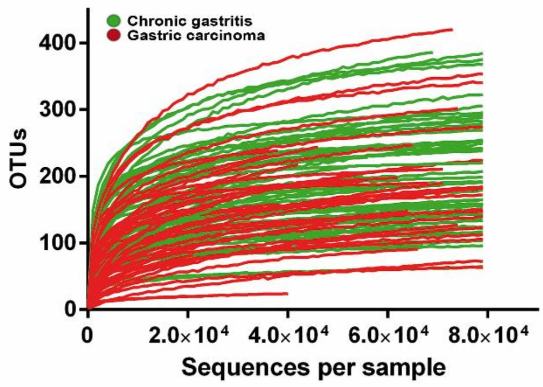

Supplementary Figure S3. Rarefaction curves of the number of OTUs versus the sequencing effort per sample. Rarefaction curves were estimated by bootstrapping of 20 random samples at 100 sequence increments to a maximum of 80,000 reads.

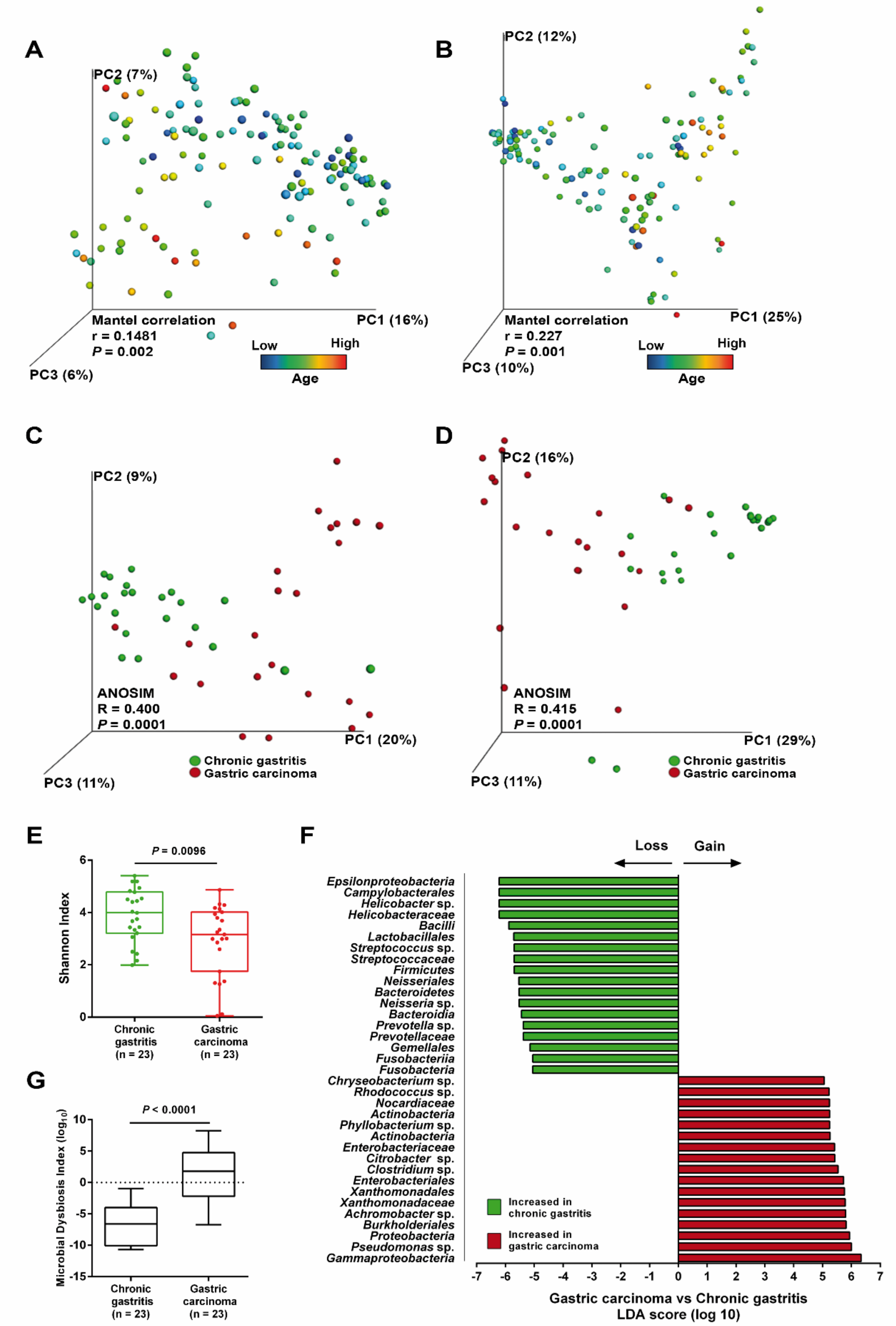

Supplementary Figure S4. PCoA plots of the unweighted **(A)** and weighted **(B)** UniFrac distances in the full sample set (discovery cohort) in which samples were coloured by increasing age. Mantel correlations controlled with 10^4 permutations were used to compare distances. PCoA plots of the unweighted **(C)** and weighted **(D)** UniFrac distances in age-matched chronic gastritis (n = 23) and

gastric carcinoma (n = 23) cases. Samples were coloured by diagnosis. Groups of patients were compared with t tests using 10^3 Monte Carlo permutations. **(E)** Shannon index of diversity among the age-matched chronic gastritis and gastric carcinoma cases. **(F)** LEfSe analysis showing the association of specific microbiota taxa with the clinical diagnosis. **(G)** Box plot showing the MDI in the age-matched chronic gastritis and gastric carcinoma cases.

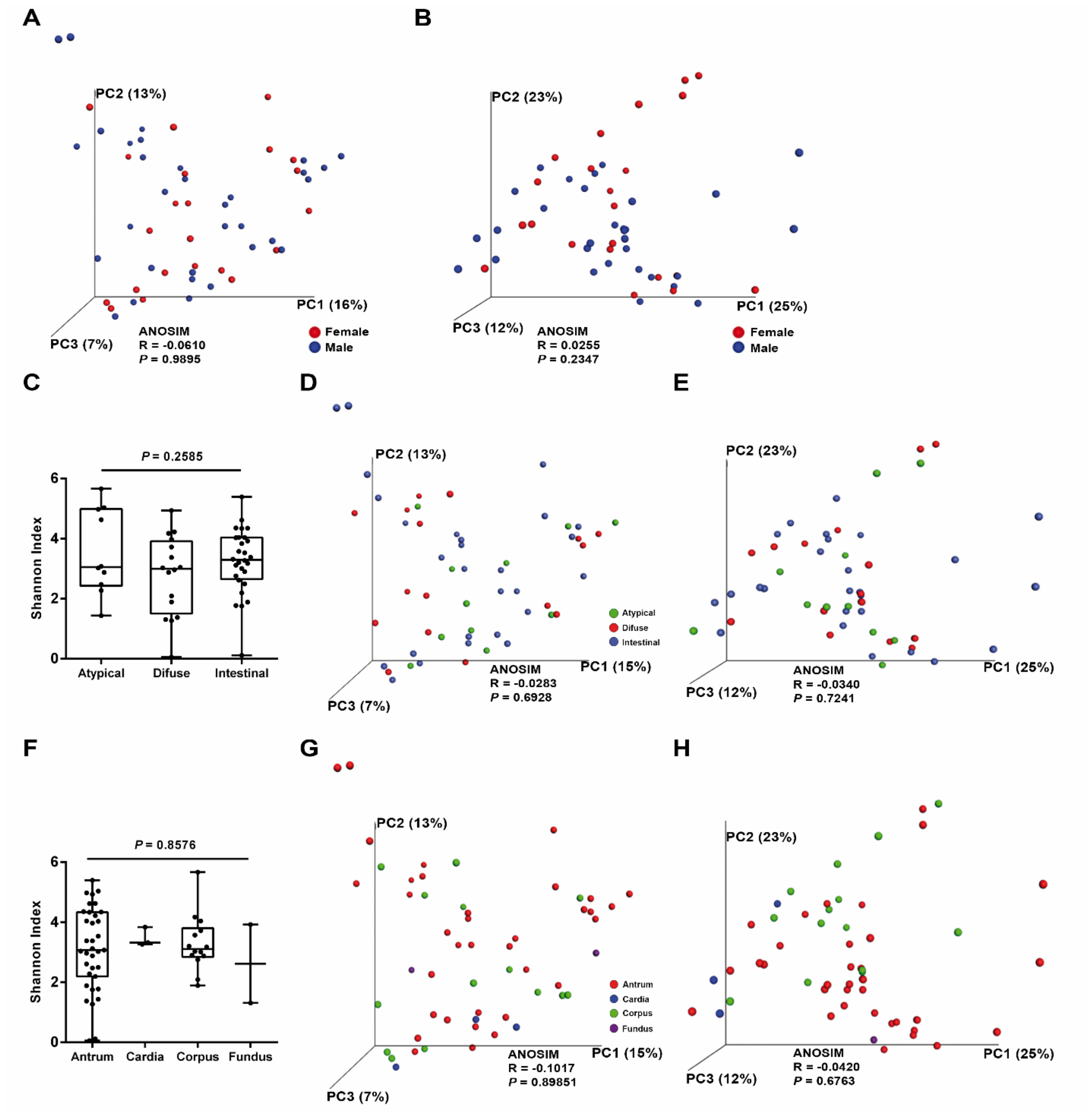

Supplementary Figure S5. (A-B) PCoA plots of the unweighted **(A)** and weighted UniFrac **(B)** distances in gastric carcinoma cases in the discovery cohort. Samples were coloured by gender. **(C)** Box plot showing the Shannon index across histological type of gastric carcinoma. **(D-E)** PCoA plots of the unweighted **(D)** and weighted **(E)** UniFrac distances in gastric carcinoma. Samples were coloured by histological type**. (F)** Box plot showing the Shannon index according to tumour location. **(G-H)** PCoA plots of the unweighted **(G)** and weighted **(H)** UniFrac distances in gastric carcinoma. Samples were coloured by tumour location.

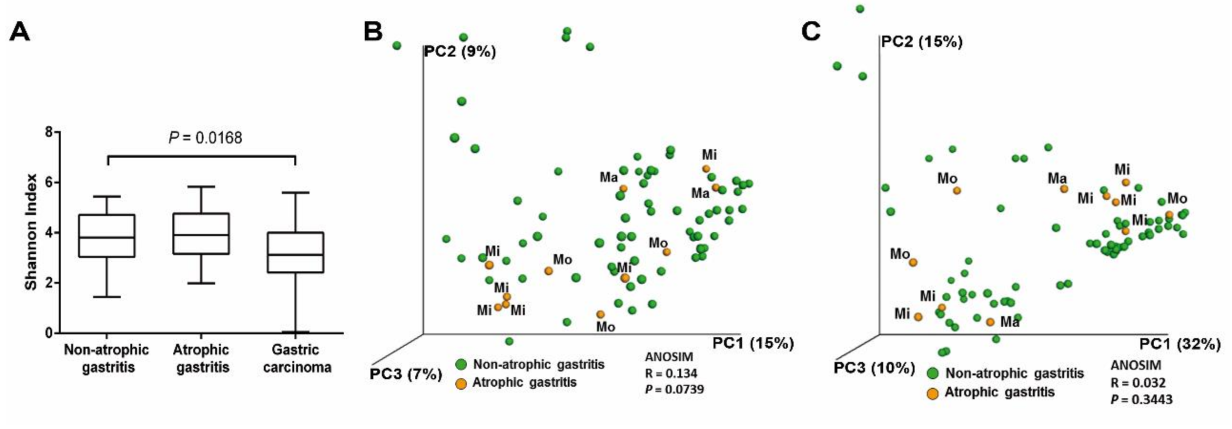

Supplementary Figure S6. (A) Boxplot showing the Shannon index of diversity in 70 non-atrophic gastritis patients, 11 patients with glandular atrophy and 54 gastric carcinoma patients. PCoA plots of the **(B)** unweighted and **(C)** weighted UniFrac distances in the group of chronic gastritis. Samples were colored by the presence of glandular atrophy. Mi, Mo, and Ma, stand for mild (n = 6), moderate (n = 3), and marked (n = 2) glandular atrophy, respectively. Gastric carcinoma samples were omitted in these plots. PC1, PC2 and PC3 represent the top three principal coordinates that captured the maximum diversity. The percentage of diversity captured by each coordinate is shown.

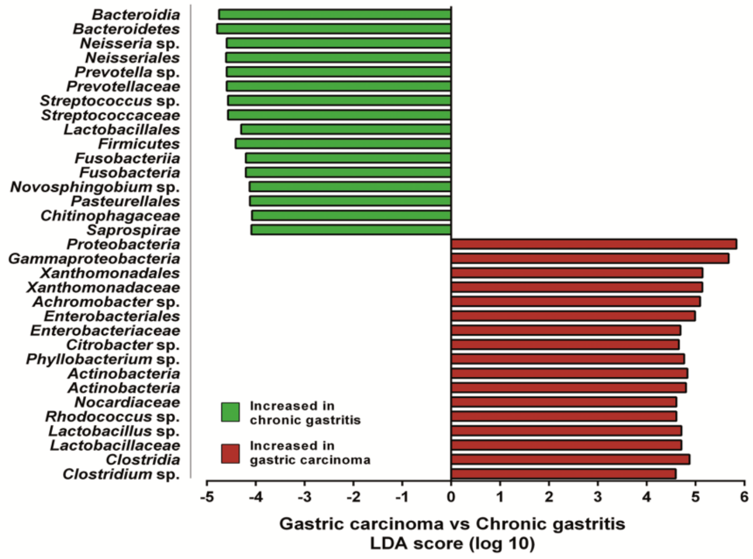

Supplementary Figure S7. LEfSe re-analysis, in which *Helicobacter* spp. reads were subtracted from the dataset. Green indicates taxa enriched in chronic gastritis group and red indicates taxa enriched in gastric carcinoma group.

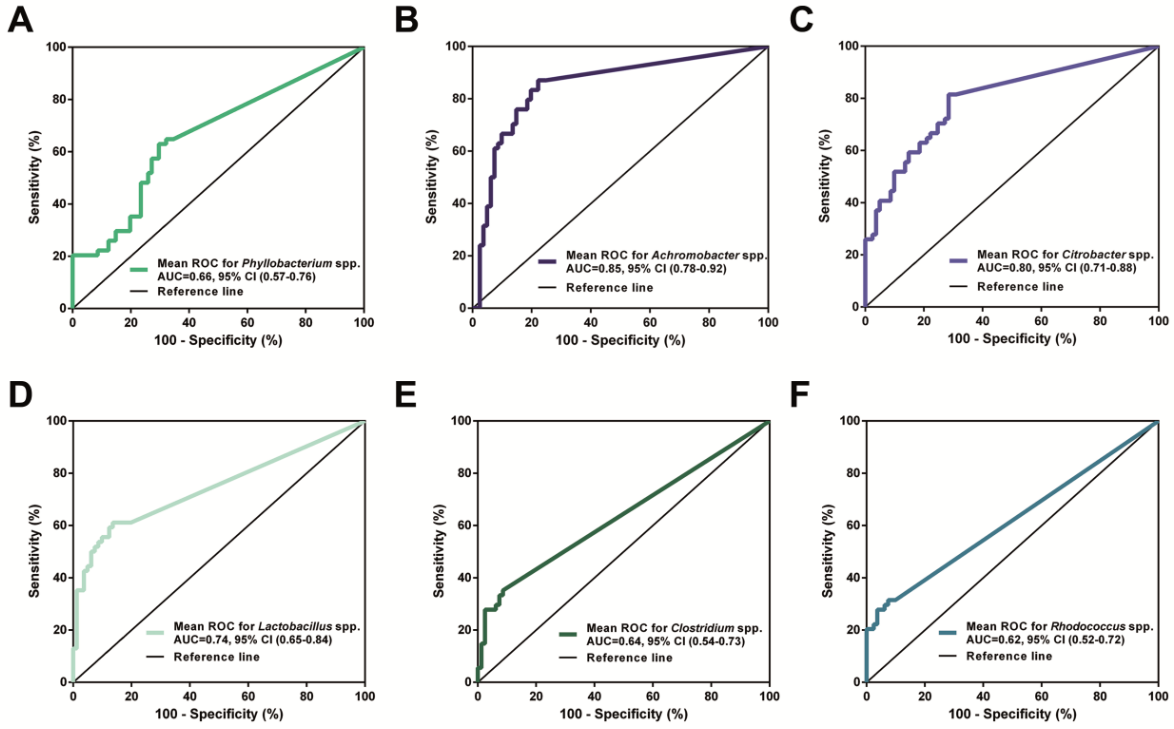

Supplementary Figure S8. Receiver operating characteristic (ROC) curves analysis to evaluate the discriminatory potential of abundance of **(A)** *Phyllobacterium* spp., **(B)** *Achromobacter* spp., **(C)** *Citrobacter* spp., **(D)** *Lactobacillus* spp., **(E)** *Clostridium* spp. and **(F)** *Rhodococcus* spp. The discriminatory potential of a biomarker was determined by the highest area under the curve.

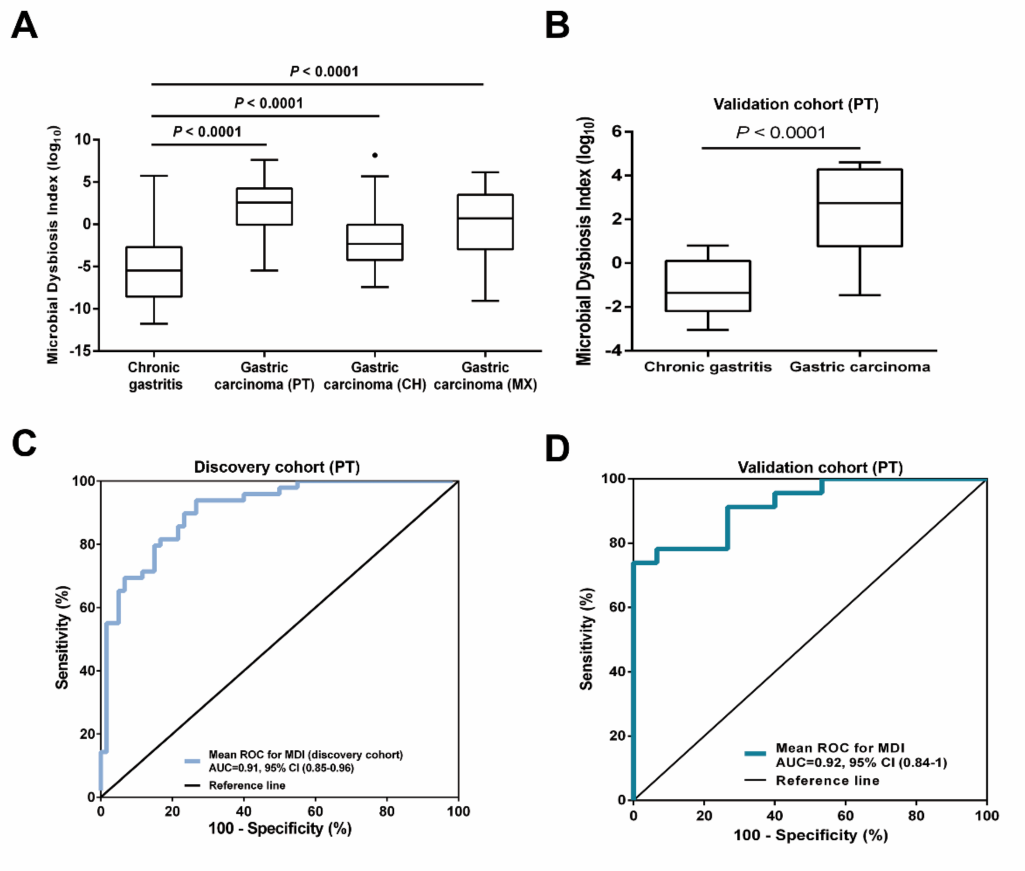

Supplementary Figure S9. Microbial dysbiosis index calculated excluding *Prevotella* sp. and *Streptococcus* sp. **(A)** Box plot showing the MDI in the discovery cohort and in the Chinese and Mexican validation cohorts. **(B)** Box plot showing the MDI the Portuguese validation cohort. Significance was obtained by Student’s t-test. **(C-D)** ROC curve analysis to evaluate the discriminatory potential of MDI in gastric carcinoma detection in the discovery cohort **(C)** and in the Portuguese validation cohort **(D).**

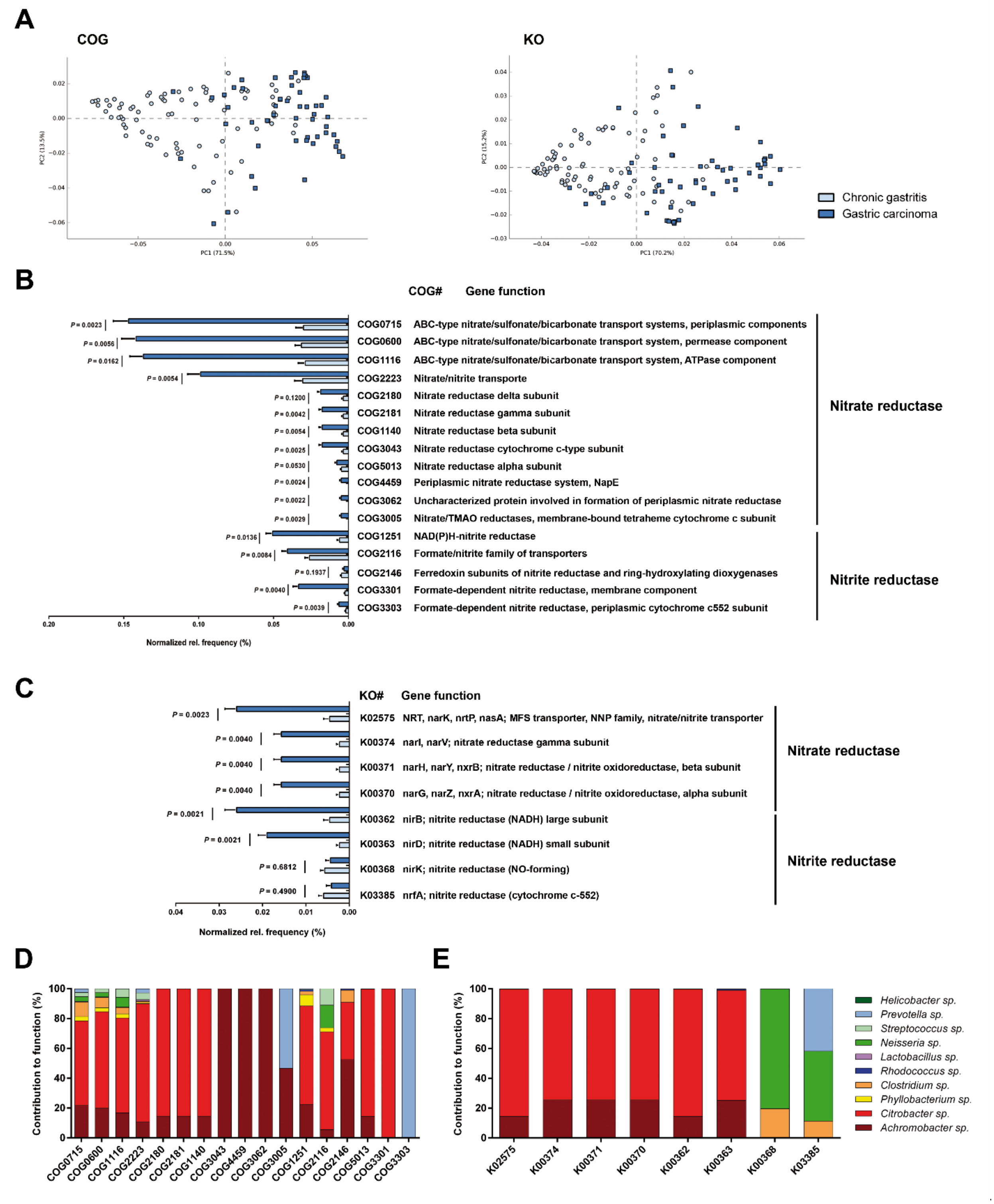

Supplementary Figure S10. (A) Principal component analysis (PCA) plot comparing the metagenome predictions (for COG and KO using PICRUSt) of the microbiota of patients with chronic gastritis and gastric carcinoma. **(B-C)** Normalized relative frequency of nitrate reductase **(B)** and nitrite reductase **(C)** functions predicted with the 10 genera differentially abundant in the two patient groups. Significance was considered for adjusted *P* < 0.05. **(D-E)** Genera contribution to each nitrate and nitrate reductase functions (Supplementary Table S5).

SUPPLEMENTARY TABLES

Supplementary Table S1. Characteristics of the patients in the Portuguese discovery and validation cohorts.

Patient characteristics	Discovery cohort			Validation cohort		
	**Chronic gastritis (n = 81)**	**Gastric carcinoma (n = 54)**	***P*** *	**Chronic gastritis (n = 15)**	**Gastric carcinoma (n = 23)**	***P*** *
Age (mean ± S.D.)	43.6 ± 7.0	58.8 ± 13.2	< 0.001	58.1 ± 16	68.9 ± 13.5	0.032
Gender (n, %)						
Male	79 (97.5)	32 (59.3)	< 0.001	5 (33.3)	16 (69.6)	0.028
Female	2 (2.5)	22 (40.7)		10 (66.7)	7 (30.4)	
H. pylori status determined by PCR-LiPA (n, %)						
Positive	80 (98.8)	47 (87.0)	0.007	-	-	
Negative	1 (1.2)	7 (13.0)		-	-	
Histological type of gastric cancer (n, %)						
Intestinal	NA	27 (50.0)		NA	11 (47.8)	
Diffuse	NA	16 (29.6)		NA	0 (0)	
Indeterminate/mixed	NA	11 (20.4)		NA	12 (52.2)	
Tumor stage (n, %)						
T1	NA	4 (7.4)		NA	1 (4.3)	
T2	NA	3 (5.6)		NA	4 (17.4)	
T3	NA	47 (87.0)		NA	12 (52.2)	
T4	NA	0 (0)		NA	6 (26.1)	
Lymph node metastasis (n, %)						
Presence	NA	44 (81.5)		NA	16 (69.6)	
Absent	NA	10 (18.5)		NA	7 (30.4)	
Vascular invasion (n, %)						
Presence	NA	28 (51.9)		NA	16 (69.6)	
Absent	NA	26 (48.1)		NA	7 (30.4)	
Distant metastasis (n, %)						
Presence	NA	1 (1.9)		NA	0 (0)	
Absent	NA	53 (98.1)		NA	23 (100)	

NA, not applicable. *P-value obtained by Fisher's exact test for categorical variables and by t test for continuous variables. Statistical tests were performed two-sided.

Supplementary Table S2. Characteristics of the patients and retrieval data in the Chinese and in the Mexican validation cohorts.

SampleID	Clinical_setting	Gender	Age	Local	SRA_Sample	SRA_Study	SRA_Project
Chi_GC_1	Gastric_carcinoma	Female	66	China	SRR3991143	SRP080738	PRJNA310127
Chi_GC_2	Gastric_carcinoma	Male	62	China	SRR3991145	SRP080738	PRJNA310127
Chi_GC_3	Gastric_carcinoma	Male	53	China	SRR3991147	SRP080738	PRJNA310127
Chi_GC_4	Gastric_carcinoma	Male	62	China	SRR3991149	SRP080738	PRJNA310127
Chi_GC_5	Gastric_carcinoma	Male	32	China	SRR3991151	SRP080738	PRJNA310127
Chi_GC_6	Gastric_carcinoma	Male	62	China	SRR3991153	SRP080738	PRJNA310127
Chi_GC_7	Gastric_carcinoma	Male	62	China	SRR3991155	SRP080738	PRJNA310127
Chi_GC_8	Gastric_carcinoma	Male	66	China	SRR3991157	SRP080738	PRJNA310127
Chi_GC_9	Gastric_carcinoma	Male	56	China	SRR3991159	SRP080738	PRJNA310127
Chi_GC_10	Gastric_carcinoma	Male	68	China	SRR3991161	SRP080738	PRJNA310127
Chi_GC_11	Gastric_carcinoma	Male	70	China	SRR3991163	SRP080738	PRJNA310127
Chi_GC_12	Gastric_carcinoma	Male	61	China	SRR3991165	SRP080738	PRJNA310127
Chi_GC_13	Gastric_carcinoma	Male	58	China	SRR3991167	SRP080738	PRJNA310127
Chi_GC_14	Gastric_carcinoma	Male	69	China	SRR3991169	SRP080738	PRJNA310127
Chi_GC_15	Gastric_carcinoma	Male	65	China	SRR3991171	SRP080738	PRJNA310127
Chi_GC_16	Gastric_carcinoma	Male	67	China	SRR3991173	SRP080738	PRJNA310127
Chi_GC_17	Gastric_carcinoma	Male	64	China	SRR3991175	SRP080738	PRJNA310127
Chi_GC_18	Gastric_carcinoma	Male	69	China	SRR3991177	SRP080738	PRJNA310127
Chi_GC_19	Gastric_carcinoma	Male	61	China	SRR3991179	SRP080738	PRJNA310127
Chi_GC_20	Gastric_carcinoma	Male	68	China	SRR3991181	SRP080738	PRJNA310127
Chi_GC_21	Gastric_carcinoma	Male	58	China	SRR3991183	SRP080738	PRJNA310127
Chi_GC_22	Gastric_carcinoma	Female	54	China	SRR3991185	SRP080738	PRJNA310127
Chi_GC_23	Gastric_carcinoma	Male	60	China	SRR3991187	SRP080738	PRJNA310127
Chi_GC_24	Gastric_carcinoma	Male	60	China	SRR3991189	SRP080738	PRJNA310127
Chi_GC_25	Gastric_carcinoma	Male	59	China	SRR3991191	SRP080738	PRJNA310127
Chi_GC_26	Gastric_carcinoma	Female	70	China	SRR3991193	SRP080738	PRJNA310127
Chi_GC_27	Gastric_carcinoma	Male	69	China	SRR3991195	SRP080738	PRJNA310127
Chi_GC_28	Gastric_carcinoma	Male	51	China	SRR3991197	SRP080738	PRJNA310127
Chi_GC_29	Gastric_carcinoma	Male	61	China	SRR3991199	SRP080738	PRJNA310127
Chi_GC_30	Gastric_carcinoma	Male	64	China	SRR3991201	SRP080738	PRJNA310127
Chi_GC_31	Gastric_carcinoma	Male	54	China	SRR3991203	SRP080738	PRJNA310127
Chi_GC_32	Gastric_carcinoma	Male	70	China	SRR3991205	SRP080738	PRJNA310127
Chi_GC_33	Gastric_carcinoma	Male	67	China	SRR3991207	SRP080738	PRJNA310127
Chi_GC_34	Gastric_carcinoma	Male	63	China	SRR3991209	SRP080738	PRJNA310127
Chi_GC_35	Gastric_carcinoma	Male	66	China	SRR3991211	SRP080738	PRJNA310127
Chi_GC_36	Gastric_carcinoma	Female	68	China	SRR3991213	SRP080738	PRJNA310127
Chi_GC_37	Gastric_carcinoma	Female	50	China	SRR3991215	SRP080738	PRJNA310127
Chi_GC_38	Gastric_carcinoma	Male	64	China	SRR3991217	SRP080738	PRJNA310127
Chi_GC_39	Gastric_carcinoma	Male	63	China	SRR3991219	SRP080738	PRJNA310127
Chi_GC_40	Gastric_carcinoma	Male	59	China	SRR3991221	SRP080738	PRJNA310127
Chi_GC_41	Gastric_carcinoma	Female	60	China	SRR3991223	SRP080738	PRJNA310127
Chi_GC_42	Gastric_carcinoma	Female	59	China	SRR3991225	SRP080738	PRJNA310127

Chi_GC_43	Gastric_carcinoma	Male	32	China	SRR3991227	SRP080738	PRJNA310127
Chi_GC_44	Gastric_carcinoma	Male	70	China	SRR3991229	SRP080738	PRJNA310127
Chi_GC_45	Gastric_carcinoma	Male	62	China	SRR3991231	SRP080738	PRJNA310127
Chi_GC_46	Gastric_carcinoma	Male	48	China	SRR3991232	SRP080738	PRJNA310127
Chi_GC_47	Gastric_carcinoma	Male	63	China	SRR3991234	SRP080738	PRJNA310127
Chi_GC_48	Gastric_carcinoma	Male	51	China	SRR3991236	SRP080738	PRJNA310127
Chi_GC_49	Gastric_carcinoma	Male	65	China	SRR3991237	SRP080738	PRJNA310127
Chi_GC_50	Gastric_carcinoma	Male	68	China	SRR3991239	SRP080738	PRJNA310127
Chi_GC_51	Gastric_carcinoma	Male	48	China	SRR3991241	SRP080738	PRJNA310127
Chi_GC_52	Gastric_carcinoma	Male	58	China	SRR3991243	SRP080738	PRJNA310127
Chi_GC_53	Gastric_carcinoma	Male	56	China	SRR3991245	SRP080738	PRJNA310127
Chi_GC_54	Gastric_carcinoma	Female	64	China	SRR3991247	SRP080738	PRJNA310127
Chi_GC_55	Gastric_carcinoma	Male	60	China	SRR3991249	SRP080738	PRJNA310127
Chi_GC_56	Gastric_carcinoma	Male	60	China	SRR3991251	SRP080738	PRJNA310127
Chi_GC_57	Gastric_carcinoma	Male	67	China	SRR3991253	SRP080738	PRJNA310127
Chi_GC_58	Gastric_carcinoma	Male	70	China	SRR3991255	SRP080738	PRJNA310127
Chi_GC_59	Gastric_carcinoma	Male	47	China	SRR3991257	SRP080738	PRJNA310127
Chi_GC_60	Gastric_carcinoma	Male	69	China	SRR3991259	SRP080738	PRJNA310127
Chi_GC_61	Gastric_carcinoma	Male	50	China	SRR3991261	SRP080738	PRJNA310127
Chi_GC_62	Gastric_carcinoma	Male	68	China	SRR3991263	SRP080738	PRJNA310127
Chi_GC_63	Gastric_carcinoma	Male	65	China	SRR3991265	SRP080738	PRJNA310127
Chi_GC_64	Gastric_carcinoma	Male	45	China	SRR3991267	SRP080738	PRJNA310127
Chi_GC_65	Gastric_carcinoma	Male	65	China	SRR3991269	SRP080738	PRJNA310127
Chi_GC_66	Gastric_carcinoma	Female	59	China	SRR3991271	SRP080738	PRJNA310127
Chi_GC_67	Gastric_carcinoma	Female	69	China	SRR3991273	SRP080738	PRJNA310127
Chi_GC_68	Gastric_carcinoma	Female	50	China	SRR3991274	SRP080738	PRJNA310127
Chi_GC_69	Gastric_carcinoma	Female	50	China	SRR3991276	SRP080738	PRJNA310127
Chi_GC_70	Gastric_carcinoma	Female	67	China	SRR3991278	SRP080738	PRJNA310127
Chi_GC_71	Gastric_carcinoma	Male	64	China	SRR3991280	SRP080738	PRJNA310127
Chi_GC_72	Gastric_carcinoma	Male	66	China	SRR3991282	SRP080738	PRJNA310127
Chi_GC_73	Gastric_carcinoma	Male	49	China	SRR3991284	SRP080738	PRJNA310127
Chi_GC_74	Gastric_carcinoma	Male	59	China	SRR3991286	SRP080738	PRJNA310127
Chi_GC_75	Gastric_carcinoma	Female	65	China	SRR3991288	SRP080738	PRJNA310127
Chi_GC_76	Gastric_carcinoma	Male	50	China	SRR3991290	SRP080738	PRJNA310127
Chi_GC_77	Gastric_carcinoma	Male	62	China	SRR3991292	SRP080738	PRJNA310127
Chi_GC_78	Gastric_carcinoma	Male	59	China	SRR3991294	SRP080738	PRJNA310127
Chi_GC_79	Gastric_carcinoma	Male	67	China	SRR3991296	SRP080738	PRJNA310127
Mex_GC_1	Gastric_carcinoma	Female	82	Mexico	SRR3991007	SRP080738	PRJNA310127
Mex_GC_2	Gastric_carcinoma	Male	72	Mexico	SRR3991010	SRP080738	PRJNA310127
Mex_GC_4	Gastric_carcinoma	Female	58	Mexico	SRR3991014	SRP080738	PRJNA310127
Mex_GC_5	Gastric_carcinoma	Female	38	Mexico	SRR3991016	SRP080738	PRJNA310127
Mex_GC_6	Gastric_carcinoma	Female	44	Mexico	SRR3991018	SRP080738	PRJNA310127
Mex_GC_7	Gastric_carcinoma	Female	70	Mexico	SRR3991020	SRP080738	PRJNA310127
Mex_GC_8	Gastric_carcinoma	Male	75	Mexico	SRR3991023	SRP080738	PRJNA310127
Mex_GC_9	Gastric_carcinoma	Female	66	Mexico	SRR3991025	SRP080738	PRJNA310127
Mex_GC_10	Gastric_carcinoma	Male	70	Mexico	SRR3991027	SRP080738	PRJNA310127
Mex_GC_11	Gastric_carcinoma	Female	56	Mexico	SRR3991028	SRP080738	PRJNA310127
Mex_GC_12	Gastric_carcinoma	Male	75	Mexico	SRR3991031	SRP080738	PRJNA310127
Mex_GC_13	Gastric_carcinoma	Female	56	Mexico	SRR3991033	SRP080738	PRJNA310127

Mex_GC_14	Gastric_carcinoma	Female	54	Mexico	SRR3991035	SRP080738	PRJNA310127
Mex_GC_15	Gastric_carcinoma	Male	69	Mexico	SRR3991038	SRP080738	PRJNA310127
Mex_GC_16	Gastric_carcinoma	Female	75	Mexico	SRR3991040	SRP080738	PRJNA310127
Mex_GC_17	Gastric_carcinoma	Female	42	Mexico	SRR3991042	SRP080738	PRJNA310127
Mex_GC_18	Gastric_carcinoma	Male	64	Mexico	SRR3991044	SRP080738	PRJNA310127
Mex_GC_19	Gastric_carcinoma	Female	52	Mexico	SRR3991046	SRP080738	PRJNA310127
Mex_GC_20	Gastric_carcinoma	Male	58	Mexico	SRR3991048	SRP080738	PRJNA310127
Mex_GC_21	Gastric_carcinoma	Female	82	Mexico	SRR3991051	SRP080738	PRJNA310127
Mex_GC_22	Gastric_carcinoma	Female	72	Mexico	SRR3991053	SRP080738	PRJNA310127
Mex_GC_23	Gastric_carcinoma	Male	57	Mexico	SRR3991056	SRP080738	PRJNA310127
Mex_GC_24	Gastric_carcinoma	Female	81	Mexico	SRR3991058	SRP080738	PRJNA310127
Mex_GC_25	Gastric_carcinoma	Male	62	Mexico	SRR3991061	SRP080738	PRJNA310127
Mex_GC_26	Gastric_carcinoma	Female	79	Mexico	SRR3991063	SRP080738	PRJNA310127
Mex_GC_27	Gastric_carcinoma	Male	38	Mexico	SRR3991066	SRP080738	PRJNA310127
Mex_GC_28	Gastric_carcinoma	Female	56	Mexico	SRR3991069	SRP080738	PRJNA310127
Mex_GC_29	Gastric_carcinoma	Female	72	Mexico	SRR3991072	SRP080738	PRJNA310127
Mex_GC_30	Gastric_carcinoma	Male	45	Mexico	SRR3991079	SRP080738	PRJNA310127
Mex_GC_31	Gastric_carcinoma	Male	69	Mexico	SRR3991082	SRP080738	PRJNA310127
Mex_GC_32	Gastric_carcinoma	Female	48	Mexico	SRR3991084	SRP080738	PRJNA310127
Mex_GC_33	Gastric_carcinoma	Male	60	Mexico	SRR3991088	SRP080738	PRJNA310127
Mex_GC_34	Gastric_carcinoma	Male	46	Mexico	SRR3991090	SRP080738	PRJNA310127
Mex_GC_35	Gastric_carcinoma	Male	56	Mexico	SRR3991092	SRP080738	PRJNA310127
Mex_GC_36	Gastric_carcinoma	Male	48	Mexico	SRR3991094	SRP080738	PRJNA310127
Mex_GC_37	Gastric_carcinoma	Male	80	Mexico	SRR3991096	SRP080738	PRJNA310127
Mex_GC_38	Gastric_carcinoma	Male	91	Mexico	SRR3991098	SRP080738	PRJNA310127
Mex_GC_39	Gastric_carcinoma	Female	56	Mexico	SRR3991100	SRP080738	PRJNA310127
Mex_GC_40	Gastric_carcinoma	Female	67	Mexico	SRR3991102	SRP080738	PRJNA310127
Mex_GC_41	Gastric_carcinoma	Male	61	Mexico	SRR3991104	SRP080738	PRJNA310127
Mex_GC_42	Gastric_carcinoma	Male	71	Mexico	SRR3991106	SRP080738	PRJNA310127
Mex_GC_43	Gastric_carcinoma	Female	77	Mexico	SRR3991110	SRP080738	PRJNA310127
Mex_GC_44	Gastric_carcinoma	Female	42	Mexico	SRR3991112	SRP080738	PRJNA310127
Mex_GC_45	Gastric_carcinoma	Female	67	Mexico	SRR3991114	SRP080738	PRJNA310127
Mex_GC_46	Gastric_carcinoma	Female	77	Mexico	SRR3991116	SRP080738	PRJNA310127
Mex_GC_47	Gastric_carcinoma	Male	37	Mexico	SRR3991118	SRP080738	PRJNA310127
Mex_GC_48	Gastric_carcinoma	Female	73	Mexico	SRR3991120	SRP080738	PRJNA310127
Mex_GC_49	Gastric_carcinoma	Female	67	Mexico	SRR3991123	SRP080738	PRJNA310127
Mex_GC_50	Gastric_carcinoma	Male	80	Mexico	SRR3991127	SRP080738	PRJNA310127
Mex_GC_51	Gastric_carcinoma	Male	76	Mexico	SRR3991129	SRP080738	PRJNA310127
Mex_GC_52	Gastric_carcinoma	Female	49	Mexico	SRR3991133	SRP080738	PRJNA310127
Mex_GC_53	Gastric_carcinoma	Male	67	Mexico	SRR3991135	SRP080738	PRJNA310127
Mex_GC_54	Gastric_carcinoma	Male	74	Mexico	SRR3991137	SRP080738	PRJNA310127

Supplementary Table S3. Primers used in qPCR and, unless otherwise stated, designed for this study.

Primer	Sequence (5'-3')	Amplicon size (bp)	Target species
Helicobacter_F<br>Helicobacter_R	GAAGATAATGACGGTATCTAAC<br>ATTTCACACCTGACTGACTAT	139	*Helicobacter* sp.
Neisseria_F<br>Neisseria_R	AACGATGTCAATTAGCTGTT<br>CAATTCCTTTGAGTTTTAATC	108	*Neisseria* sp.
Achromo_F1<br>Achromo_R1	TCGGGCCTTGGTAGCG<br>TTCCTTTGAGTTTTAATCTT	77	*Achromobacter* sp.
Phyllo_F<br>Phyllo_R	CTGCCTTTGATACTGGTAGT<br>CGGCTAGCTCTCATAGTTTA	202	*Phyllobacterium* sp.
Clostr_F*<br>Clostr_R*	ATGCAAGTCGAGCGAKG<br>TATGCGGTATTAATCTKCCTTT	120	*Clostridium* sp.
Rhodo_F<br>Rhodo_R	GGGTTCCTTCCACGGGAT<br>CCTTTGAGTTTTAGCCTTG	84	*Rhodococcus* sp.
Lactob2_F<br>Lactob2_R	GAGGCAGCAGTAGGGAATCTTC<br>GGCCAGTTACTACCTCTATCCTTCTTC	126	*Lactobacillus* sp.
Citro F1<br>Citro R2	GTAAAGTACTTTCAGCGAG<br>GTTTCGGATGCAGTTCCC	216	*Citrobacter* sp.
Prevo_F<br>Prevo_R	CACGGTAAACGATGGATGCC<br>CAATTCCTTTGAGTTTCACC	113	*Prevotella* sp.
Strepto_F<br>Strepto_R	TGTCGTGAGATGTTGGGTTAAG<br>CCACCTTCCTCCGGTTTATTAC	112	*Streptococcus* sp.
340F§<br>515R§	TCCTACGGGAGGCAGCAGT<br>CGTATTACCGCGGCTGCTGGCAC	198	Universal

*Rinttila T *et al.*, Journal of Applied Microbiology 2004.[8]

§Horz HP *et al.*, Journal of Clinical Microbiology 2005.[9]

Supplementary Table S4. Quality control results of 32 gastric samples derived from 16 subjects of the discovery set distributed across amplification and sequencing sets*.

Microbiota measurement	Intraclass correlation coefficient[#] ICC (95% CI)	*P*[§]
**Alpha diversity (Shannon index)**	0.70 (-0.23 – 0.92)	< 0.0001
**Beta diversity[&]**		
Unweighted UniFrac	0.67 (-0.05 – 0.89)	0.003
Weighted UniFrac	0.91 (0.64 – 0.97)	< 0.0001
**Relative abundance of major phyla**		
Proteobacteria	0.69 (0.07 – 0.89)	0.004
Firmicutes	0.62 (-0.05 – 0.86)	0.034
Bacteroidetes	0.66 (0.07 – 0.88)	0.01
Fusobacteria	0.73 (0.27 – 0.91)	0.005

ICC, Intraclass correlation coefficient; CI, confidence intervals.

*The 16S rRNA reads were analyzed using the UPARSE pipeline applying the same quality filtering parameters for each set of sequencing (maximum expected errors of 0.50 and global trimming of 250nt).

[#]Intraclass correlation coefficients were computed assuming the two-way mixed average measures model and using the absolute agreement definition. Higher ICC and lower *P*-value indicate better reproducibility.

[§]*P*-values obtained by analysis of variance (ANOVA) for the proportion of variance estimated for between subjects.

[&]First principal coordinate of weighted UniFrac distance matrix.

Supplementary Table S5. Correlations between the relative abundance of *Helicobacter* spp. and other phyla.

**Taxa**	**Chronic gastritis (ρ* and *P* - value)**	**Gastric carcinoma (ρ* and *P* - value)**
Non-*Helicobacter Proteobacteria*	-0.59 (*P* < 0.0001)	-0.14 (*P* = 0.2640)
Firmicutes	-0.49 (*P* < 0.0001)	0.05 (*P* = 0.6889)
Bacteroidetes	-0.43 (*P* < 0.0001)	0.48 (*P* = 0.0003)
Actinobacteria	-0.54 (*P* < 0.0001)	-0.17 (*P* = 0.2405)
Fusobacteria	-0.15 (*P* = 0.1750)	0.29 (*P* = 0.0352)

*ρ, Spearman's rank coefficient.

Supplementary Table S6. Predicted KEGG Pathways differentially abundant between chronic gastritis and gastric carcinoma.

KEGG_Pathways	Chronic gastritis mean rel. freq. (%)	Gastric carcinoma mean rel. freq. (%)	*P*-values (corrected)*	LDA score	*P*-value (LEfSe)[#]	Enriched Category
Environmental Information Processing|Membrane Transport	10.92161	15.12360	0.00819	4.33586	1.44E-13	GC
Metabolism|Carbohydrate Metabolism	9.03294	9.83316	0.00178	3.61487	8.98E-05	GC
Genetic Information Processing|Transcription	1.88669	2.63088	0.00273	3.59340	4.64E-16	GC
Metabolism|Xenobiotics Biodegradation and Metabolism	2.50552	3.28664	0.00216	3.56750	1.54E-06	GC
Unclassified|Cellular Processes and Signaling	3.70073	4.38594	0.00186	3.53616	2.97E-09	GC
Unclassified|Poorly Characterized	4.57739	5.11194	0.00241	3.44255	3.58E-11	GC
Unclassified|Metabolism	2.19640	2.66576	0.00410	3.38182	6.97E-12	GC
Environmental Information Processing|Signal Transduction	1.97292	2.39848	0.00315	3.32954	4.67E-07	GC
Metabolism|Amino Acid Metabolism	9.43605	9.81096	0.04352	3.23132	1.19E-02	GC
Metabolism|Lipid Metabolism	3.03535	3.37183	0.00228	3.22117	1.87E-05	GC
Metabolism|Biosynthesis of Other Secondary Metabolites	0.61655	0.75324	0.02048	2.86853	3.31E-08	GC
Metabolism|Enzyme Families	1.79516	1.91412	0.00282	2.86758	3.75E-04	GC
Metabolism|Metabolism of Other Amino Acids	1.77550	1.87891	0.00410	2.77646	7.74E-05	GC
Organismal Systems|Excretory System	0.01130	0.02874	0.01365	2.67809	7.84E-11	GC
Genetic Information Processing|Replication and Repair	8.79776	6.76323	0.00195	4.00196	1.32E-12	CGa
Genetic Information Processing|Translation	5.77603	4.16490	0.00256	3.89954	2.36E-12	CGa
Metabolism|Energy Metabolism	6.03451	5.00229	0.00146	3.72885	1.63E-16	CGa
Unclassified|Genetic Information Processing	3.25102	2.34838	0.00171	3.65858	2.24E-15	CGa
Cellular Processes|Cell Motility	3.79669	2.98665	0.00455	3.64273	1.26E-03	CGa
Metabolism|Glycan Biosynthesis and Metabolism	2.80951	1.96937	0.00341	3.62993	4.66E-14	CGa
Metabolism|Nucleotide Metabolism	3.92975	3.13489	0.04096	3.59115	3.17E-11	CGa
Genetic Information Processing|Folding, Sorting and Degradation	2.82319	2.24511	0.00158	3.47981	1.33E-14	CGa
Metabolism|Metabolism of Cofactors and Vitamins	4.34925	3.85149	0.00293	3.41535	4.34E-12	CGa
Human Diseases|Neurodegenerative Diseases	0.52178	0.32498	0.00585	3.11480	1.09E-10	CGa
Organismal Systems|Circulatory System	0.10186	0.04917	0.00152	2.97205	5.55E-10	CGa
Cellular Processes|Cell Growth and Death	0.59027	0.42469	0.01024	2.96612	8.95E-13	CGa
Human Diseases|Metabolic Diseases	0.07488	0.07169	0.29446	2.91240	4.92E-02	CGa
Human Diseases|Cancers	0.17859	0.13235	0.00205	2.80085	2.31E-08	CGa

Environmental Information Processing|Signaling Molecules and Interaction	0.29797	0.20385	0.00372	2.76556	1.05E-11	CGa
Organismal Systems|Environmental Adaptation	0.17243	0.13526	0.00164	2.70978	3.76E-08	CGa
Human Diseases|Immune System Diseases	0.06094	0.04869	0.00683	2.69694	3.57E-08	CGa
Organismal Systems|Immune System	0.07854	0.05444	0.00512	2.63043	4.56E-07	CGa
Organismal Systems|Endocrine System	0.24091	0.25913	0.29240	-	-	-
Cellular Processes|Cell Communication	0.00000	0.00000	1.02500	-	-	-
Metabolism|Metabolism of Terpenoids and Polyketides	1.77672	1.77557	1.03658	-	-	-
Organismal Systems|Sensory System	0.00000	0.00000	1.00000	-	-	-
Human Diseases|Cardiovascular Diseases	0.00841	0.01131	0.15661	-	-	-
Human Diseases|Infectious Diseases	0.51979	0.49543	0.13653	-	-	-
Cellular Processes|Transport and Catabolism	0.24157	0.23500	0.57989	-	-	-
Organismal Systems|Nervous System	0.07261	0.08275	0.02510	-	-	-
Organismal Systems|Digestive System	0.03089	0.03518	0.18139	-	-	-

*White's non-parametric t-test corrected with Benjaminin-Hochberg FDR
#Wilcoxon test

Supplementary Table S7 (A-F). Predicted metagenome of the gastric microbiota using clusters of orthologous groups (COG) in PICRUSt.[4] Predicted COG and KO functions were analysed in STAMP[5] using the two-group comparison with White's non-parametric *t* test[6] and corrected for multiple test with Benjamini-Hochberg false discovery rate. The accuracy of the predicted metagenomes was assessed by determining the NSTI value for each microbiome sample. **Panel A**, contains the selected COG and KO functions predicted with the whole bacterial community; **Panel B**, contains the weighted NSTI values for each microbiome sample predicted (COG functions) with the whole bacterial community; **Panel C**, contains the weighted NSTI values for each microbiome sample predicted (KO functions) with the whole bacterial community; **Panel D**, contains the selected COG and KO functions predicted with 10 genera differentially abundant in the two patient groups (by LEfSe analysis); **Panel E**, contains the weighted NSTI values for each microbiome sample predicted (COG functions) with 10 genera differentially abundant in the two patient groups (by LEfSe analysis); **Panel F**, contains the weighted NSTI values for each microbiome sample predicted (KO functions) with 10 genera differentially abundant in the two patient groups (by LEfSe analysis).

Panel A.

Selected COG functions predicted with the whole bacterial community

#COG	Chronic_gastritis: mean rel. freq. (%)	Chronic_gastritis: std. dev. (%)	Gastric_carcinoma: mean rel. freq. (%)	Gastric_carcinoma: std. dev. (%)	p-values (corrected)	Difference between means	95.0% lower CI	95.0% upper CI
COG0600	0.0380803377962	0.0409023940591	0.144434615098	0.0623153699431	0.00232614809874	-0.106354277302	-0.125221671315	-0.0858149024638
COG0715	0.0511934133291	0.0458494571997	0.157705490347	0.0545866953999	0.00562539692974	-0.106512077018	-0.123411161547	-0.0869708850513
COG1116	0.0367227762667	0.0375718046599	0.134767467249	0.0547153422806	0.0162278399567	-0.0980446909824	-0.113905723223	-0.0821855767254
COG1140	0.0025440844066	0.00668464772303	0.0155970995959	0.0148806082199	0.00415556665557	-0.0130530151893	-0.017275905303	-0.00882205712479
COG1251	0.011611977352	0.0151913533865	0.0519315230767	0.0265861055036	0.0137960022686	-0.0403195457247	-0.0485243035945	-0.0319322400119
COG2116	0.0233649620796	0.0215994256599	0.0383015898969	0.0250015856269	0.00383590768206	-0.0149366278173	-0.0224787062647	-0.00737941715869
COG2146	0.00873193589374	0.0106158185296	0.0393208615594	0.0228568326087	0.00404667184042	-0.0305889256656	-0.0370139696261	-0.0238474856576
COG2180	0.0025440844066	0.00668464772303	0.0155970995959	0.0148806082199	0.00246255801811	-0.0130530151893	-0.0174246808685	-0.0088974980323
COG2181	0.0025440844066	0.00668464772303	0.0155970995959	0.0148806082199	0.00536082059038	-0.0130530151893	-0.0172555501534	-0.00875565238332
COG2223	0.0325291097158	0.0393469296998	0.102362968402	0.0569046880422	0.00538494126796	-0.0698338586861	-0.0868249683248	-0.0530108581266
COG3005	0.00488371320348	0.00661558452324	0.0058839829874	0.00724089219848	0.545794864476	-0.00100026978392	-0.00336821329968	0.00138511644863
COG3043	0.000775554391147	0.00136327433874	0.00373812723423	0.00526742345456	0.00291015974907	-0.00296257284309	-0.00449609407818	-0.001519306695
COG3062	0.000775554391147	0.00136327433874	0.00373812723423	0.00526742345456	0.00240080882007	-0.00296257284309	-0.00452292313383	-0.00156887470169
COG3301	0.00110220332143	0.00305540177519	0.00558613612147	0.00725633566029	0.00396292449273	-0.00448393280004	-0.00645199014736	-0.00259824027501
COG3303	0.00410815881234	0.00672591354877	0.00214585575317	0.0048409549419	0.0601975829892	0.00196230305917	0.0000265626740328	0.00375532751947
COG4459	0.000775554391147	0.00136327433874	0.00373812723423	0.00526742345456	0.00220101737343	-0.00296257284309	-0.00452548718722	-0.00152154912066
COG5013	0.00254435706316	0.00668457771702	0.016518260486	0.0143514761835	0.136777508206	-0.0139739034229	-0.0180142674333	-0.00970549825398

Selected KO functions predicted with the whole bacterial community

#KO	Chronic_gastritis: mean rel. freq. (%)	Chronic_gastritis: std. dev. (%)	Gastric_carcinoma: mean rel. freq. (%)	Gastric_carcinoma: std. dev. (%)	p-values (corrected)	Difference between means	95.0% lower CI	95.0% upper CI
K02575	0.019212388	0.017022566	0.038842644	0.016814508	0.002251173	-0.019630256	-0.025259151	-0.013779889
K00374	0.005812651	0.006131977	0.011933115	0.009218057	0.0037737	-0.006120464	-0.00876988	-0.003022912
K00371	0.003691426	0.004422298	0.011473263	0.009304943	0.003769578	-0.007781837	-0.01046599	-0.005165154
K00370	0.00585703	0.006274888	0.011669446	0.009237582	0.003767521	-0.005812416	-0.00887716	-0.003032478
K00362	0.015170144	0.014939699	0.029964508	0.018805713	0.002091545	-0.014794365	-0.020454654	-0.008977207
K00363	0.013528508	0.013678413	0.021447784	0.013696811	0.002090911	-0.007919277	-0.01298771	-0.003372292

K00368	0.006984151	0.007328579	0.012471795	0.012708158	0.011156408	-0.005487644	-0.009290866	-0.001638056
K03385	0.00473815	0.006007843	0.002046396	0.00567851	0.011201674	0.002691754	0.000679126	0.004591805

Panel B.

Weighted NSTI values for each microbiome sample predicted with the whole bacterial community: COG functions			
GC1	Gastric_carcinoma	Weighted NSTI	0.040489631851
GC2	Gastric_carcinoma	Weighted NSTI	0.0264613580275
GC3	Gastric_carcinoma	Weighted NSTI	0.0389247982662
GC4	Gastric_carcinoma	Weighted NSTI	0.0269396264775
GC5	Gastric_carcinoma	Weighted NSTI	0.0643272483964
GC6	Gastric_carcinoma	Weighted NSTI	0.034023649827
GC7	Gastric_carcinoma	Weighted NSTI	0.033400643341
GC8	Gastric_carcinoma	Weighted NSTI	0.0358000414837
GC9	Gastric_carcinoma	Weighted NSTI	0.093279905086
GC10	Gastric_carcinoma	Weighted NSTI	0.0936946331635
GC11	Gastric_carcinoma	Weighted NSTI	0.0366245943864
GC12	Gastric_carcinoma	Weighted NSTI	0.0287537167868
GC13	Gastric_carcinoma	Weighted NSTI	0.0382200425585
GC14	Gastric_carcinoma	Weighted NSTI	0.0362654260905
GC15	Gastric_carcinoma	Weighted NSTI	0.0472873255435
GC16	Gastric_carcinoma	Weighted NSTI	0.0307910984753
GC17	Gastric_carcinoma	Weighted NSTI	0.0383074646772
GC18	Gastric_carcinoma	Weighted NSTI	0.0147998010024
GC19	Gastric_carcinoma	Weighted NSTI	0.0250345494043
GC20	Gastric_carcinoma	Weighted NSTI	0.0196118356432
GC21	Gastric_carcinoma	Weighted NSTI	0.0554188523192
GC22	Gastric_carcinoma	Weighted NSTI	0.0185227799536
GC23	Gastric_carcinoma	Weighted NSTI	0.119390269582
GC24	Gastric_carcinoma	Weighted NSTI	0.0354898499502
GC25	Gastric_carcinoma	Weighted NSTI	0.0288768762561
GC26	Gastric_carcinoma	Weighted NSTI	0.0200459069109
GC27	Gastric_carcinoma	Weighted NSTI	0.0224176229194
GC28	Gastric_carcinoma	Weighted NSTI	0.0310864469154
GC29	Gastric_carcinoma	Weighted NSTI	0.0584871720662
GC30	Gastric_carcinoma	Weighted NSTI	0.0380997257276
GC31	Gastric_carcinoma	Weighted NSTI	0.0199768652024
GC32	Gastric_carcinoma	Weighted NSTI	0.0326920914222
GC33	Gastric_carcinoma	Weighted NSTI	0.0793141787269
GC34	Gastric_carcinoma	Weighted NSTI	0.0408301561178
GC35	Gastric_carcinoma	Weighted NSTI	0.174612125786
GC36	Gastric_carcinoma	Weighted NSTI	0.0367613957595
GC37	Gastric_carcinoma	Weighted NSTI	0.0346428244292
GC38	Gastric_carcinoma	Weighted NSTI	0.0271150607156
GC39	Gastric_carcinoma	Weighted NSTI	0.0344307993816
GC40	Gastric_carcinoma	Weighted NSTI	0.0666759514741
GC41	Gastric_carcinoma	Weighted NSTI	0.0335315244906
GC42	Gastric_carcinoma	Weighted NSTI	0.030448097136
GC43	Gastric_carcinoma	Weighted NSTI	0.0747453733925
GC44	Gastric_carcinoma	Weighted NSTI	0.0283546264849
GC45	Gastric_carcinoma	Weighted NSTI	0.0428279962603
GC46	Gastric_carcinoma	Weighted NSTI	0.0377074018348
GC47	Gastric_carcinoma	Weighted NSTI	0.0460050016118
GC48	Gastric_carcinoma	Weighted NSTI	0.0517707933249
GC49	Gastric_carcinoma	Weighted NSTI	0.0147181524782
GC50	Gastric_carcinoma	Weighted NSTI	0.0264651673989
GC51	Gastric_carcinoma	Weighted NSTI	0.0310639160311
GC52	Gastric_carcinoma	Weighted NSTI	0.0282792295803
GC53	Gastric_carcinoma	Weighted NSTI	0.0753222820128
GC54	Gastric_carcinoma	Weighted NSTI	0.0394247749183
Ga1	Chronic_gastritis	Weighted NSTI	0.0908111634672
Ga2	Chronic_gastritis	Weighted NSTI	0.0346916585769
Ga3	Chronic_gastritis	Weighted NSTI	0.117402392327
Ga4	Chronic_gastritis	Weighted NSTI	0.121956578849
Ga5	Chronic_gastritis	Weighted NSTI	0.0443341197835
Ga6	Chronic_gastritis	Weighted NSTI	0.0564023880138
Ga7	Chronic_gastritis	Weighted NSTI	0.057121902691
Ga8	Chronic_gastritis	Weighted NSTI	0.0283503088713
Ga9	Chronic_gastritis	Weighted NSTI	0.122618605685
Ga10	Chronic_gastritis	Weighted NSTI	0.137675241471
Ga11	Chronic_gastritis	Weighted NSTI	0.0313511145222
Ga12	Chronic_gastritis	Weighted NSTI	0.0359385545363
Ga13	Chronic_gastritis	Weighted NSTI	0.0267607348016
Ga14	Chronic_gastritis	Weighted NSTI	0.0399913286286
Ga15	Chronic_gastritis	Weighted NSTI	0.0334904226307
Ga16	Chronic_gastritis	Weighted NSTI	0.0555738114619
Ga17	Chronic_gastritis	Weighted NSTI	0.0590495787144

Ga18	Chronic_gastritis	Weighted NSTI	0.0310918907492
Ga19	Chronic_gastritis	Weighted NSTI	0.0381330828939
Ga20	Chronic_gastritis	Weighted NSTI	0.030050458735
Ga21	Chronic_gastritis	Weighted NSTI	0.0293018480457
Ga22	Chronic_gastritis	Weighted NSTI	0.0406953226685
Ga23	Chronic_gastritis	Weighted NSTI	0.0319070615753
Ga24	Chronic_gastritis	Weighted NSTI	0.0305824730562
Ga25	Chronic_gastritis	Weighted NSTI	0.0317728785598
Ga26	Chronic_gastritis	Weighted NSTI	0.0270866195057
Ga27	Chronic_gastritis	Weighted NSTI	0.0313910654489
Ga28	Chronic_gastritis	Weighted NSTI	0.0258875683418
Ga29	Chronic_gastritis	Weighted NSTI	0.0313598318794
Ga30	Chronic_gastritis	Weighted NSTI	0.0711050885032
Ga31	Chronic_gastritis	Weighted NSTI	0.0244993143869
Ga32	Chronic_gastritis	Weighted NSTI	0.024942786618
Ga33	Chronic_gastritis	Weighted NSTI	0.030719788708
Ga34	Chronic_gastritis	Weighted NSTI	0.0261586219593
Ga35	Chronic_gastritis	Weighted NSTI	0.0304169841173
Ga36	Chronic_gastritis	Weighted NSTI	0.0313967257136
Ga37	Chronic_gastritis	Weighted NSTI	0.0256214740952
Ga38	Chronic_gastritis	Weighted NSTI	0.0406025576612
Ga39	Chronic_gastritis	Weighted NSTI	0.0236312029641
Ga40	Chronic_gastritis	Weighted NSTI	0.0265096285674
Ga41	Chronic_gastritis	Weighted NSTI	0.0235599356195
Ga42	Chronic_gastritis	Weighted NSTI	0.0326502167956
Ga43	Chronic_gastritis	Weighted NSTI	0.0316268011843
Ga44	Chronic_gastritis	Weighted NSTI	0.0243789299692
Ga45	Chronic_gastritis	Weighted NSTI	0.0248214547776
Ga46	Chronic_gastritis	Weighted NSTI	0.0263703541274
Ga47	Chronic_gastritis	Weighted NSTI	0.0236490670651
Ga48	Chronic_gastritis	Weighted NSTI	0.0419295596547
Ga49	Chronic_gastritis	Weighted NSTI	0.0453555338905
Ga50	Chronic_gastritis	Weighted NSTI	0.0395148501086
Ga51	Chronic_gastritis	Weighted NSTI	0.0367415424425
Ga52	Chronic_gastritis	Weighted NSTI	0.0434132457438
Ga53	Chronic_gastritis	Weighted NSTI	0.0290510505889
Ga54	Chronic_gastritis	Weighted NSTI	0.0522665861212
Ga55	Chronic_gastritis	Weighted NSTI	0.0404586124022
Ga56	Chronic_gastritis	Weighted NSTI	0.0412643460406
Ga57	Chronic_gastritis	Weighted NSTI	0.0402077309941
Ga58	Chronic_gastritis	Weighted NSTI	0.0253999624615
Ga59	Chronic_gastritis	Weighted NSTI	0.0428094549349
Ga60	Chronic_gastritis	Weighted NSTI	0.0378768132663
Ga61	Chronic_gastritis	Weighted NSTI	0.0503554281182
Ga62	Chronic_gastritis	Weighted NSTI	0.0485291220322
Ga63	Chronic_gastritis	Weighted NSTI	0.0490305079779
Ga64	Chronic_gastritis	Weighted NSTI	0.0934052073583
Ga65	Chronic_gastritis	Weighted NSTI	0.0346860076556
Ga66	Chronic_gastritis	Weighted NSTI	0.0463552549761
Ga67	Chronic_gastritis	Weighted NSTI	0.0302419700077
Ga68	Chronic_gastritis	Weighted NSTI	0.0357172222252
Ga69	Chronic_gastritis	Weighted NSTI	0.0237149281501
Ga70	Chronic_gastritis	Weighted NSTI	0.0448817341401
Ga71	Chronic_gastritis	Weighted NSTI	0.0256301408615
Ga72	Chronic_gastritis	Weighted NSTI	0.0387136886567
Ga73	Chronic_gastritis	Weighted NSTI	0.038742864283
Ga74	Chronic_gastritis	Weighted NSTI	0.031060976654
Ga75	Chronic_gastritis	Weighted NSTI	0.0333810306831
Ga76	Chronic_gastritis	Weighted NSTI	0.0252564503647
Ga77	Chronic_gastritis	Weighted NSTI	0.0296099257201
Ga78	Chronic_gastritis	Weighted NSTI	0.0797687144484
Ga79	Chronic_gastritis	Weighted NSTI	0.055401172199
Ga80	Chronic_gastritis	Weighted NSTI	0.0282654611359
Ga81	Chronic_gastritis	Weighted NSTI	0.060914477945

Panel C.

Weighted NSTI values for each microbiome sample predicted with the whole bacterial community: KO functions			
#Sample	Clinical setting	Metric	Value
GC1	Gastric_carcinoma	Weighted NSTI	0.040489631851
GC2	Gastric_carcinoma	Weighted NSTI	0.0264613580275
GC3	Gastric_carcinoma	Weighted NSTI	0.0389247982662
GC4	Gastric_carcinoma	Weighted NSTI	0.0269396264775
GC5	Gastric_carcinoma	Weighted NSTI	0.0643272483964
GC6	Gastric_carcinoma	Weighted NSTI	0.034023649827
GC7	Gastric_carcinoma	Weighted NSTI	0.033400643341
GC8	Gastric_carcinoma	Weighted NSTI	0.0358000414837
GC9	Gastric_carcinoma	Weighted NSTI	0.093279905086

GC10	Gastric_carcinoma	Weighted NSTI	0.0936946331635
GC11	Gastric_carcinoma	Weighted NSTI	0.0366245943864
GC12	Gastric_carcinoma	Weighted NSTI	0.0287537167868
GC13	Gastric_carcinoma	Weighted NSTI	0.0382200425585
GC14	Gastric_carcinoma	Weighted NSTI	0.0362654260905
GC15	Gastric_carcinoma	Weighted NSTI	0.0472873255435
GC16	Gastric_carcinoma	Weighted NSTI	0.0307910984753
GC17	Gastric_carcinoma	Weighted NSTI	0.0383074646772
GC18	Gastric_carcinoma	Weighted NSTI	0.0147998010024
GC19	Gastric_carcinoma	Weighted NSTI	0.0250345494043
GC20	Gastric_carcinoma	Weighted NSTI	0.0196118356432
GC21	Gastric_carcinoma	Weighted NSTI	0.0554188523192
GC22	Gastric_carcinoma	Weighted NSTI	0.0185227799536
GC23	Gastric_carcinoma	Weighted NSTI	0.119390269582
GC24	Gastric_carcinoma	Weighted NSTI	0.0354898499502
GC25	Gastric_carcinoma	Weighted NSTI	0.0288768762561
GC26	Gastric_carcinoma	Weighted NSTI	0.0200459069109
GC27	Gastric_carcinoma	Weighted NSTI	0.0224176229194
GC28	Gastric_carcinoma	Weighted NSTI	0.0310864469154
GC29	Gastric_carcinoma	Weighted NSTI	0.0584871720662
GC30	Gastric_carcinoma	Weighted NSTI	0.0380997257276
GC31	Gastric_carcinoma	Weighted NSTI	0.0199768652024
GC32	Gastric_carcinoma	Weighted NSTI	0.0326920914222
GC33	Gastric_carcinoma	Weighted NSTI	0.0793141787269
GC34	Gastric_carcinoma	Weighted NSTI	0.0408301561178
GC35	Gastric_carcinoma	Weighted NSTI	0.174612125786
GC36	Gastric_carcinoma	Weighted NSTI	0.0367613957595
GC37	Gastric_carcinoma	Weighted NSTI	0.0346428244292
GC38	Gastric_carcinoma	Weighted NSTI	0.0271150607156
GC39	Gastric_carcinoma	Weighted NSTI	0.0344307993816
GC40	Gastric_carcinoma	Weighted NSTI	0.0666759514741
GC41	Gastric_carcinoma	Weighted NSTI	0.0335315244906
GC42	Gastric_carcinoma	Weighted NSTI	0.030448097136
GC43	Gastric_carcinoma	Weighted NSTI	0.0747453733925
GC44	Gastric_carcinoma	Weighted NSTI	0.0283546264849
GC45	Gastric_carcinoma	Weighted NSTI	0.0428279962603
GC46	Gastric_carcinoma	Weighted NSTI	0.0377074018348
GC47	Gastric_carcinoma	Weighted NSTI	0.0460050016118
GC48	Gastric_carcinoma	Weighted NSTI	0.0517707933249
GC49	Gastric_carcinoma	Weighted NSTI	0.0147181524782
GC50	Gastric_carcinoma	Weighted NSTI	0.0264651673989
GC51	Gastric_carcinoma	Weighted NSTI	0.0310639160311
GC52	Gastric_carcinoma	Weighted NSTI	0.0282792295803
GC53	Gastric_carcinoma	Weighted NSTI	0.0753222820128
GC54	Gastric_carcinoma	Weighted NSTI	0.0394247749183
Ga1	Chronic_gastritis	Weighted NSTI	0.0908111634672
Ga2	Chronic_gastritis	Weighted NSTI	0.0346916585769
Ga3	Chronic_gastritis	Weighted NSTI	0.117402392327
Ga4	Chronic_gastritis	Weighted NSTI	0.121956578849
Ga5	Chronic_gastritis	Weighted NSTI	0.0443341197835
Ga6	Chronic_gastritis	Weighted NSTI	0.0564023880138
Ga7	Chronic_gastritis	Weighted NSTI	0.057121902691
Ga8	Chronic_gastritis	Weighted NSTI	0.0283503088713
Ga9	Chronic_gastritis	Weighted NSTI	0.122618605685
Ga10	Chronic_gastritis	Weighted NSTI	0.137675241471
Ga11	Chronic_gastritis	Weighted NSTI	0.0313511145222
Ga12	Chronic_gastritis	Weighted NSTI	0.0359385545363
Ga13	Chronic_gastritis	Weighted NSTI	0.0267607348016
Ga14	Chronic_gastritis	Weighted NSTI	0.0399913286286
Ga15	Chronic_gastritis	Weighted NSTI	0.0334904226307
Ga16	Chronic_gastritis	Weighted NSTI	0.0555738114619
Ga17	Chronic_gastritis	Weighted NSTI	0.0590495787144
Ga18	Chronic_gastritis	Weighted NSTI	0.0310918907492
Ga19	Chronic_gastritis	Weighted NSTI	0.0381330828939
Ga20	Chronic_gastritis	Weighted NSTI	0.030050458735
Ga21	Chronic_gastritis	Weighted NSTI	0.0293018480457
Ga22	Chronic_gastritis	Weighted NSTI	0.0406953226685
Ga23	Chronic_gastritis	Weighted NSTI	0.0319070615753
Ga24	Chronic_gastritis	Weighted NSTI	0.0305824730562
Ga25	Chronic_gastritis	Weighted NSTI	0.0317728785598
Ga26	Chronic_gastritis	Weighted NSTI	0.0270866195057
Ga27	Chronic_gastritis	Weighted NSTI	0.0313910654489
Ga28	Chronic_gastritis	Weighted NSTI	0.0258875683418
Ga29	Chronic_gastritis	Weighted NSTI	0.0313598318794
Ga30	Chronic_gastritis	Weighted NSTI	0.0711050885032
Ga31	Chronic_gastritis	Weighted NSTI	0.0244993143869
Ga32	Chronic_gastritis	Weighted NSTI	0.024942786618
Ga33	Chronic_gastritis	Weighted NSTI	0.030719788708
Ga34	Chronic_gastritis	Weighted NSTI	0.0261586219593
Ga35	Chronic_gastritis	Weighted NSTI	0.0304169841173
Ga36	Chronic_gastritis	Weighted NSTI	0.0313967257136

Ga37	Chronic_gastritis	Weighted NSTI	0.0256214740952
Ga38	Chronic_gastritis	Weighted NSTI	0.0406025576612
Ga39	Chronic_gastritis	Weighted NSTI	0.0236312029641
Ga40	Chronic_gastritis	Weighted NSTI	0.0265096285674
Ga41	Chronic_gastritis	Weighted NSTI	0.0235599356195
Ga42	Chronic_gastritis	Weighted NSTI	0.0326502167956
Ga43	Chronic_gastritis	Weighted NSTI	0.0316268011843
Ga44	Chronic_gastritis	Weighted NSTI	0.0243789299692
Ga45	Chronic_gastritis	Weighted NSTI	0.0248214547776
Ga46	Chronic_gastritis	Weighted NSTI	0.0263703541274
Ga47	Chronic_gastritis	Weighted NSTI	0.0236490670651
Ga48	Chronic_gastritis	Weighted NSTI	0.0419295596547
Ga49	Chronic_gastritis	Weighted NSTI	0.0453555338905
Ga50	Chronic_gastritis	Weighted NSTI	0.0395148501086
Ga51	Chronic_gastritis	Weighted NSTI	0.0367415424425
Ga52	Chronic_gastritis	Weighted NSTI	0.0434132457438
Ga53	Chronic_gastritis	Weighted NSTI	0.0290510505889
Ga54	Chronic_gastritis	Weighted NSTI	0.0522665861212
Ga55	Chronic_gastritis	Weighted NSTI	0.0404586124022
Ga56	Chronic_gastritis	Weighted NSTI	0.0412643460406
Ga57	Chronic_gastritis	Weighted NSTI	0.0402077309941
Ga58	Chronic_gastritis	Weighted NSTI	0.0253999624615
Ga59	Chronic_gastritis	Weighted NSTI	0.0428094549349
Ga60	Chronic_gastritis	Weighted NSTI	0.0378768132663
Ga61	Chronic_gastritis	Weighted NSTI	0.0503554281182
Ga62	Chronic_gastritis	Weighted NSTI	0.0485291220322
Ga63	Chronic_gastritis	Weighted NSTI	0.0490305079779
Ga64	Chronic_gastritis	Weighted NSTI	0.0934052073583
Ga65	Chronic_gastritis	Weighted NSTI	0.0346860076556
Ga66	Chronic_gastritis	Weighted NSTI	0.0463552549761
Ga67	Chronic_gastritis	Weighted NSTI	0.0302419700077
Ga68	Chronic_gastritis	Weighted NSTI	0.0357172222252
Ga69	Chronic_gastritis	Weighted NSTI	0.0237149281501
Ga70	Chronic_gastritis	Weighted NSTI	0.0448817341401
Ga71	Chronic_gastritis	Weighted NSTI	0.0256301408615
Ga72	Chronic_gastritis	Weighted NSTI	0.0387136886567
Ga73	Chronic_gastritis	Weighted NSTI	0.038742864283
Ga74	Chronic_gastritis	Weighted NSTI	0.031060976654
Ga75	Chronic_gastritis	Weighted NSTI	0.0333810306831
Ga76	Chronic_gastritis	Weighted NSTI	0.0252564503647
Ga77	Chronic_gastritis	Weighted NSTI	0.0296099257201
Ga78	Chronic_gastritis	Weighted NSTI	0.0797687144484
Ga79	Chronic_gastritis	Weighted NSTI	0.055401172199
Ga80	Chronic_gastritis	Weighted NSTI	0.0282654611359
Ga81	Chronic_gastritis	Weighted NSTI	0.060914477945

Panel D.

#COG	Chronic_gastritis mean rel. freq. (%)	Chronic_gastritis std. dev. (%)	Gastric_carcinoma mean rel. freq. (%)	Gastric_carcinoma std. dev. (%)	p-values (corrected)	Difference between means	95.0% lower CI	95.0% upper CI
COG0600	0.0300418645333	0.0460843980869	0.146812141835	0.0737182608186	0.00231043088186	-0.116770277302	-0.138788727797	-0.0945946985164
COG0715	0.0314438736083	0.0378427117516	0.141752334688	0.0701383419265	0.00565196314901	-0.11030846108	-0.130485067169	-0.0895309924737
COG1116	0.0287626358989	0.0418888860315	0.136926410484	0.066415304397	0.0161185615731	-0.108163774585	-0.128837496949	-0.0887058740264
COG1140	0.00341973019684	0.00933044303473	0.0174785528014	0.0151631416482	0.00413046832374	-0.0140588226046	-0.0187097703159	-0.00942096154861
COG1251	0.00588295135724	0.0139110162012	0.0505727873507	0.0317275825981	0.0136387828696	-0.0446898359934	-0.0547299434599	-0.0351107891734
COG2116	0.0260441305425	0.0264772369147	0.0406912022371	0.0264159913953	0.00839272929034	-0.0146470716946	-0.0239204671983	-0.00526594122529
COG2146	0.00216100378478	0.00477317428429	0.0333160685549	0.0270121916546	0.00401949016559	-0.0311550647701	-0.0387130722049	-0.0241121414691
COG2180	0.00341973019684	0.00933044303473	0.0174785528014	0.0151631416482	0.00244995536705	-0.0140588226046	-0.0185814422875	-0.00920331912275
COG2181	0.00341973019684	0.00933044303473	0.0174785528014	0.0151631416482	0.00539100539101	-0.0140588226046	-0.0184239543674	-0.00958313672034
COG2223	0.0304687792229	0.04819766388	0.0987132679002	0.0636281509332	0.00541539908056	-0.0682444886773	-0.0886157069943	-0.0463657519206
COG3005	0.0045568813437	0.00692789916142	0.0076798560699	0.00846065593	0.052708949082	-0.00312297472628	-0.00583622152869	-0.000214930074913
COG3043	0.000004459489807	0.00001909505648	0.004824471628	0.00652515766495	0.00289782856369	-0.00482001213819	-0.00673429348284	-0.00305935378494
COG3062	0.000004459489807	0.00001909505648	0.004824471628	0.00652515766495	0.00238169790409	-0.00482001213819	-0.00659843293099	-0.00315031401884
COG3301	0.00170781910463	0.00466443438518	0.0063267980526	0.00731620351516	0.00393361773805	-0.00461897894799	-0.00686032626101	-0.00248275954591
COG3303	0.0045524218539	0.00692771582096	0.0028553844419	0.00594717363718	0.193659849192	0.00169703741191	-0.000513423867157	0.00393403320397
COG4459	0.000004459489807	0.00001909505648	0.004824471628	0.00652515766495	0.00218694051494	-0.00482001213819	-0.00662053015558	-0.00317972339968
COG5013	0.00342002704444	0.00933034241022	0.0184927461628	0.0144801404877	0.11968031968	-0.0150727191183	-0.0194776299944	-0.010939489594

#KO	Chronic_gastritis mean rel. freq. (%)	Chronic_gastritis std. dev. (%)	Gastric_carcinoma mean rel. freq. (%)	Gastric_carcinoma std. dev. (%)	p-values (corrected)	Difference between means	95.0% lower CI	95.0% upper CI
K02575	0.004509995	0.012372823	0.025966323	0.019687365	0.002297636	-0.021456328	-0.027262001	-0.01542714
K00374	0.002257451	0.006187414	0.01563650	0.012771472	0.004038676	-0.013379049	-0.017113515	-0.009667895
K00371	0.002257451	0.006187414	0.01563650	0.012771472	0.004036315	-0.013379049	-0.016970983	-0.009580145
K00370	0.002257451	0.006187414	0.01563650	0.012771472	0.004033956	-0.013379049	-0.016896677	-0.009447808
K00362	0.004510228	0.012372741	0.025965619	0.019687411	0.002109443	-0.021455391	-0.027549823	-0.015594188
K00363	0.002258658	0.006187010	0.018956808	0.014490694	0.002108799	-0.016698150	-0.020845732	-0.012818462
K00368	0.005625786	0.008819855	0.004356963	0.007688918	0.681206315	0.001268823	-0.001531841	0.004088364
K03385	0.005919455	0.010112995	0.004099644	0.008672612	0.489792805	0.001819811	-0.001240937	0.004759555

Panel E.

Weighted NSTI values for each microbiome sample predicted with 10 genera differentially abundant in the two patient groups (by LEfSe analysis): COG functions			
#Sample	Clinical setting	Metric	Value
GC1	Gastric_carcinoma	Weighted NSTI	0.00930809485847
GC2	Gastric_carcinoma	Weighted NSTI	0.0240044368429
GC3	Gastric_carcinoma	Weighted NSTI	0.0109010814095
GC4	Gastric_carcinoma	Weighted NSTI	0.0145213951541
GC5	Gastric_carcinoma	Weighted NSTI	0.023718870231
GC6	Gastric_carcinoma	Weighted NSTI	0.0293275862309
GC7	Gastric_carcinoma	Weighted NSTI	0.00497999715557
GC8	Gastric_carcinoma	Weighted NSTI	0.023024085928
GC9	Gastric_carcinoma	Weighted NSTI	0.0937727775039
GC10	Gastric_carcinoma	Weighted NSTI	0.0938399144357
GC11	Gastric_carcinoma	Weighted NSTI	0.0231372989755
GC12	Gastric_carcinoma	Weighted NSTI	0.0102467154304
GC13	Gastric_carcinoma	Weighted NSTI	0.0345476705214
GC14	Gastric_carcinoma	Weighted NSTI	0.00921013087128
GC15	Gastric_carcinoma	Weighted NSTI	0.0414846156894
GC16	Gastric_carcinoma	Weighted NSTI	0.0423956274665
GC17	Gastric_carcinoma	Weighted NSTI	0.0154562656542
GC18	Gastric_carcinoma	Weighted NSTI	0.0136105222154
GC19	Gastric_carcinoma	Weighted NSTI	0.0134520120571
GC20	Gastric_carcinoma	Weighted NSTI	0.0139521366017
GC21	Gastric_carcinoma	Weighted NSTI	0.0645339594707
GC22	Gastric_carcinoma	Weighted NSTI	0.0133382328625
GC23	Gastric_carcinoma	Weighted NSTI	0.0354155953905
GC24	Gastric_carcinoma	Weighted NSTI	0.0208627090301
GC25	Gastric_carcinoma	Weighted NSTI	0.0181078888419
GC26	Gastric_carcinoma	Weighted NSTI	0.0144427683349
GC27	Gastric_carcinoma	Weighted NSTI	0.0160166205041
GC28	Gastric_carcinoma	Weighted NSTI	0.0233542930185
GC29	Gastric_carcinoma	Weighted NSTI	0.025739929714
GC30	Gastric_carcinoma	Weighted NSTI	0.0113294205463
GC31	Gastric_carcinoma	Weighted NSTI	0.0148387168242
GC32	Gastric_carcinoma	Weighted NSTI	0.0260766868078
GC33	Gastric_carcinoma	Weighted NSTI	0.0804474660945
GC34	Gastric_carcinoma	Weighted NSTI	0.0157898849295
GC35	Gastric_carcinoma	Weighted NSTI	0.0367311418522
GC36	Gastric_carcinoma	Weighted NSTI	0.0141705107879
GC37	Gastric_carcinoma	Weighted NSTI	0.032360819261
GC38	Gastric_carcinoma	Weighted NSTI	0.011977852744
GC39	Gastric_carcinoma	Weighted NSTI	0.0181153823328
GC40	Gastric_carcinoma	Weighted NSTI	0.0262472709806
GC41	Gastric_carcinoma	Weighted NSTI	0.0164562967724
GC42	Gastric_carcinoma	Weighted NSTI	0.0154662402134
GC43	Gastric_carcinoma	Weighted NSTI	0.0381879853417
GC44	Gastric_carcinoma	Weighted NSTI	0.0160909987093
GC45	Gastric_carcinoma	Weighted NSTI	0.0218091017727
GC46	Gastric_carcinoma	Weighted NSTI	0.0277717197922
GC47	Gastric_carcinoma	Weighted NSTI	0.0368600029383
GC48	Gastric_carcinoma	Weighted NSTI	0.0162168230646
GC49	Gastric_carcinoma	Weighted NSTI	0.0152481813312
GC50	Gastric_carcinoma	Weighted NSTI	0.0220793569132
GC51	Gastric_carcinoma	Weighted NSTI	0.0233230434466
GC52	Gastric_carcinoma	Weighted NSTI	0.0201161357312
GC53	Gastric_carcinoma	Weighted NSTI	0.0202110531638
GC54	Gastric_carcinoma	Weighted NSTI	0.0414352900421
Ga1	Chronic_gastritis	Weighted NSTI	0.0308221809121
Ga2	Chronic_gastritis	Weighted NSTI	0.0190175746876
Ga3	Chronic_gastritis	Weighted NSTI	0.0222307471725
Ga4	Chronic_gastritis	Weighted NSTI	0.0209507992895
Ga5	Chronic_gastritis	Weighted NSTI	0.0319685746606
Ga6	Chronic_gastritis	Weighted NSTI	0.0153921588649
Ga7	Chronic_gastritis	Weighted NSTI	0.0217206904368
Ga8	Chronic_gastritis	Weighted NSTI	0.0232925028465
Ga9	Chronic_gastritis	Weighted NSTI	0.0247355306637
Ga10	Chronic_gastritis	Weighted NSTI	0.0213686407209
Ga11	Chronic_gastritis	Weighted NSTI	0.0233840454215
Ga12	Chronic_gastritis	Weighted NSTI	0.0229725174811
Ga13	Chronic_gastritis	Weighted NSTI	0.0236147972795
Ga14	Chronic_gastritis	Weighted NSTI	0.0188148415636
Ga15	Chronic_gastritis	Weighted NSTI	0.0239628754086
Ga16	Chronic_gastritis	Weighted NSTI	0.0258857082524
Ga17	Chronic_gastritis	Weighted NSTI	0.0266591487112
Ga18	Chronic_gastritis	Weighted NSTI	0.0234975410995
Ga19	Chronic_gastritis	Weighted NSTI	0.0275340254447
Ga20	Chronic_gastritis	Weighted NSTI	0.0265223213943
Ga21	Chronic_gastritis	Weighted NSTI	0.0237405518119
Ga22	Chronic_gastritis	Weighted NSTI	0.0246237461481
Ga23	Chronic_gastritis	Weighted NSTI	0.0261776323705
Ga24	Chronic_gastritis	Weighted NSTI	0.0240216954059
Ga25	Chronic_gastritis	Weighted NSTI	0.0216813543149
Ga26	Chronic_gastritis	Weighted NSTI	0.0245844643959
Ga27	Chronic_gastritis	Weighted NSTI	0.0236851326843
Ga28	Chronic_gastritis	Weighted NSTI	0.0235497859161
Ga29	Chronic_gastritis	Weighted NSTI	0.0276284179621
Ga30	Chronic_gastritis	Weighted NSTI	0.0324789141807
Ga31	Chronic_gastritis	Weighted NSTI	0.0235609158646
Ga32	Chronic_gastritis	Weighted NSTI	0.0234512561062
Ga33	Chronic_gastritis	Weighted NSTI	0.0258031842255
Ga34	Chronic_gastritis	Weighted NSTI	0.0239299087937
Ga35	Chronic_gastritis	Weighted NSTI	0.0276010614863
Ga36	Chronic_gastritis	Weighted NSTI	0.0250065251942
Ga37	Chronic_gastritis	Weighted NSTI	0.0233063614292
Ga38	Chronic_gastritis	Weighted NSTI	0.0321749195517

Ga39	Chronic_gastritis	Weighted NSTI	0.0233674455307
Ga40	Chronic_gastritis	Weighted NSTI	0.0239810959362
Ga41	Chronic_gastritis	Weighted NSTI	0.0231499448874
Ga42	Chronic_gastritis	Weighted NSTI	0.0232485466453
Ga43	Chronic_gastritis	Weighted NSTI	0.0265209393083
Ga44	Chronic_gastritis	Weighted NSTI	0.0235484717435
Ga45	Chronic_gastritis	Weighted NSTI	0.0236658097207
Ga46	Chronic_gastritis	Weighted NSTI	0.0234256131336
Ga47	Chronic_gastritis	Weighted NSTI	0.023129553295
Ga48	Chronic_gastritis	Weighted NSTI	0.0248635470616
Ga49	Chronic_gastritis	Weighted NSTI	0.0238738216243
Ga50	Chronic_gastritis	Weighted NSTI	0.0234692827327
Ga51	Chronic_gastritis	Weighted NSTI	0.0284928431634
Ga52	Chronic_gastritis	Weighted NSTI	0.0232210470076
Ga53	Chronic_gastritis	Weighted NSTI	0.0241237951842
Ga54	Chronic_gastritis	Weighted NSTI	0.0249728754939
Ga55	Chronic_gastritis	Weighted NSTI	0.0245525155737
Ga56	Chronic_gastritis	Weighted NSTI	0.0247968261668
Ga57	Chronic_gastritis	Weighted NSTI	0.0220259722126
Ga58	Chronic_gastritis	Weighted NSTI	0.0235104559057
Ga59	Chronic_gastritis	Weighted NSTI	0.0340624095354
Ga60	Chronic_gastritis	Weighted NSTI	0.0248388022169
Ga61	Chronic_gastritis	Weighted NSTI	0.0371165514265
Ga62	Chronic_gastritis	Weighted NSTI	0.0315506630709
Ga63	Chronic_gastritis	Weighted NSTI	0.0190374080374
Ga64	Chronic_gastritis	Weighted NSTI	0.0255534901151
Ga65	Chronic_gastritis	Weighted NSTI	0.0283541207743
Ga66	Chronic_gastritis	Weighted NSTI	0.0243016108554
Ga67	Chronic_gastritis	Weighted NSTI	0.0251248866666
Ga68	Chronic_gastritis	Weighted NSTI	0.0329422136093
Ga69	Chronic_gastritis	Weighted NSTI	0.0234692839506
Ga70	Chronic_gastritis	Weighted NSTI	0.031955855137
Ga71	Chronic_gastritis	Weighted NSTI	0.0231236853239
Ga72	Chronic_gastritis	Weighted NSTI	0.0316024332108
Ga73	Chronic_gastritis	Weighted NSTI	0.0290991240903
Ga74	Chronic_gastritis	Weighted NSTI	0.0228351266279
Ga75	Chronic_gastritis	Weighted NSTI	0.0244748764283
Ga76	Chronic_gastritis	Weighted NSTI	0.0233080136803
Ga77	Chronic_gastritis	Weighted NSTI	0.0232002651225
Ga78	Chronic_gastritis	Weighted NSTI	0.0236748666552
Ga79	Chronic_gastritis	Weighted NSTI	0.0408731774542
Ga80	Chronic_gastritis	Weighted NSTI	0.0233605857388
Ga81	Chronic_gastritis	Weighted NSTI	0.0286796217287

Panel F.

Weighted NSTI values for each microbiome sample predicted with 10 genera differentially abundant in the two patient groups (by LEfSe analysis): KO functions			
#Sample	Clinical setting	Metric	Value
GC1	Gastric_carcinoma	Weighted NSTI	0.00930809485847
GC2	Gastric_carcinoma	Weighted NSTI	0.0240044368429
GC3	Gastric_carcinoma	Weighted NSTI	0.0109010814095
GC4	Gastric_carcinoma	Weighted NSTI	0.0145213951541
GC5	Gastric_carcinoma	Weighted NSTI	0.023718870231
GC6	Gastric_carcinoma	Weighted NSTI	0.0293275862309
GC7	Gastric_carcinoma	Weighted NSTI	0.00497999715557
GC8	Gastric_carcinoma	Weighted NSTI	0.023024085928
GC9	Gastric_carcinoma	Weighted NSTI	0.0937727775039
GC10	Gastric_carcinoma	Weighted NSTI	0.0938399144357
GC11	Gastric_carcinoma	Weighted NSTI	0.0231372989755
GC12	Gastric_carcinoma	Weighted NSTI	0.0102467154304
GC13	Gastric_carcinoma	Weighted NSTI	0.0345476705214
GC14	Gastric_carcinoma	Weighted NSTI	0.00921013087128
GC15	Gastric_carcinoma	Weighted NSTI	0.0414846156894
GC16	Gastric_carcinoma	Weighted NSTI	0.0423956274665
GC17	Gastric_carcinoma	Weighted NSTI	0.0154562656542
GC18	Gastric_carcinoma	Weighted NSTI	0.0136105222154
GC19	Gastric_carcinoma	Weighted NSTI	0.0134520120571
GC20	Gastric_carcinoma	Weighted NSTI	0.0139521366017
GC21	Gastric_carcinoma	Weighted NSTI	0.0645339594707
GC22	Gastric_carcinoma	Weighted NSTI	0.0133382328625
GC23	Gastric_carcinoma	Weighted NSTI	0.0354155953905
GC24	Gastric_carcinoma	Weighted NSTI	0.0208627090301
GC25	Gastric_carcinoma	Weighted NSTI	0.0181078888419
GC26	Gastric_carcinoma	Weighted NSTI	0.0144427683349
GC27	Gastric_carcinoma	Weighted NSTI	0.0160166205041
GC28	Gastric_carcinoma	Weighted NSTI	0.0233542930185
GC29	Gastric_carcinoma	Weighted NSTI	0.025739929714
GC30	Gastric_carcinoma	Weighted NSTI	0.0113294205463
GC31	Gastric_carcinoma	Weighted NSTI	0.0148387168242
GC32	Gastric_carcinoma	Weighted NSTI	0.0260766868078
GC33	Gastric_carcinoma	Weighted NSTI	0.0804474660945
GC34	Gastric_carcinoma	Weighted NSTI	0.0157898849295
GC35	Gastric_carcinoma	Weighted NSTI	0.0367311418522
GC36	Gastric_carcinoma	Weighted NSTI	0.0141705107879
GC37	Gastric_carcinoma	Weighted NSTI	0.032360819261

GC38	Gastric_carcinoma	Weighted NSTI	0.011977852744
GC39	Gastric_carcinoma	Weighted NSTI	0.0181153823328
GC40	Gastric_carcinoma	Weighted NSTI	0.0262472709806
GC41	Gastric_carcinoma	Weighted NSTI	0.0164562967724
GC42	Gastric_carcinoma	Weighted NSTI	0.0154662402134
GC43	Gastric_carcinoma	Weighted NSTI	0.0381879853417
GC44	Gastric_carcinoma	Weighted NSTI	0.0160909987093
GC45	Gastric_carcinoma	Weighted NSTI	0.0218091017727
GC46	Gastric_carcinoma	Weighted NSTI	0.0277717197922
GC47	Gastric_carcinoma	Weighted NSTI	0.0368600029383
GC48	Gastric_carcinoma	Weighted NSTI	0.0162168230646
GC49	Gastric_carcinoma	Weighted NSTI	0.0152481813312
GC50	Gastric_carcinoma	Weighted NSTI	0.0220793569132
GC51	Gastric_carcinoma	Weighted NSTI	0.0233230434466
GC52	Gastric_carcinoma	Weighted NSTI	0.0201161357312
GC53	Gastric_carcinoma	Weighted NSTI	0.0202110531638
GC54	Gastric_carcinoma	Weighted NSTI	0.0414352900421
Ga1	Chronic_gastritis	Weighted NSTI	0.0308221809121
Ga2	Chronic_gastritis	Weighted NSTI	0.0190175746876
Ga3	Chronic_gastritis	Weighted NSTI	0.0222307471725
Ga4	Chronic_gastritis	Weighted NSTI	0.0209507992895
Ga5	Chronic_gastritis	Weighted NSTI	0.0319685746606
Ga6	Chronic_gastritis	Weighted NSTI	0.0153921588649
Ga7	Chronic_gastritis	Weighted NSTI	0.0217206904368
Ga8	Chronic_gastritis	Weighted NSTI	0.0232925028465
Ga9	Chronic_gastritis	Weighted NSTI	0.0247355306637
Ga10	Chronic_gastritis	Weighted NSTI	0.0213686407209
Ga11	Chronic_gastritis	Weighted NSTI	0.0233840454215
Ga12	Chronic_gastritis	Weighted NSTI	0.0229725174811
Ga13	Chronic_gastritis	Weighted NSTI	0.0236147972795
Ga14	Chronic_gastritis	Weighted NSTI	0.0188148415636
Ga15	Chronic_gastritis	Weighted NSTI	0.0239628754086
Ga16	Chronic_gastritis	Weighted NSTI	0.0258857082524
Ga17	Chronic_gastritis	Weighted NSTI	0.0266591487112
Ga18	Chronic_gastritis	Weighted NSTI	0.0234975410995
Ga19	Chronic_gastritis	Weighted NSTI	0.0275340254447
Ga20	Chronic_gastritis	Weighted NSTI	0.0265223213943
Ga21	Chronic_gastritis	Weighted NSTI	0.0237405518119
Ga22	Chronic_gastritis	Weighted NSTI	0.0246237461481
Ga23	Chronic_gastritis	Weighted NSTI	0.0261776323705
Ga24	Chronic_gastritis	Weighted NSTI	0.0240216954059
Ga25	Chronic_gastritis	Weighted NSTI	0.0216813543149
Ga26	Chronic_gastritis	Weighted NSTI	0.0245844643959
Ga27	Chronic_gastritis	Weighted NSTI	0.0236851326843
Ga28	Chronic_gastritis	Weighted NSTI	0.0235497859161
Ga29	Chronic_gastritis	Weighted NSTI	0.0276284179621
Ga30	Chronic_gastritis	Weighted NSTI	0.0324789141807
Ga31	Chronic_gastritis	Weighted NSTI	0.0235609158646
Ga32	Chronic_gastritis	Weighted NSTI	0.0234512561062
Ga33	Chronic_gastritis	Weighted NSTI	0.0258031842255
Ga34	Chronic_gastritis	Weighted NSTI	0.0239299087937
Ga35	Chronic_gastritis	Weighted NSTI	0.0276010614863
Ga36	Chronic_gastritis	Weighted NSTI	0.0250065251942
Ga37	Chronic_gastritis	Weighted NSTI	0.0233063614292
Ga38	Chronic_gastritis	Weighted NSTI	0.0321749195517
Ga39	Chronic_gastritis	Weighted NSTI	0.0233674455307
Ga40	Chronic_gastritis	Weighted NSTI	0.0239810959362
Ga41	Chronic_gastritis	Weighted NSTI	0.0231499448874
Ga42	Chronic_gastritis	Weighted NSTI	0.0232485466453
Ga43	Chronic_gastritis	Weighted NSTI	0.0265209393083
Ga44	Chronic_gastritis	Weighted NSTI	0.0235484717435
Ga45	Chronic_gastritis	Weighted NSTI	0.0236658097207
Ga46	Chronic_gastritis	Weighted NSTI	0.0234256131336
Ga47	Chronic_gastritis	Weighted NSTI	0.023129553295
Ga48	Chronic_gastritis	Weighted NSTI	0.0248635470616
Ga49	Chronic_gastritis	Weighted NSTI	0.0238738216243
Ga50	Chronic_gastritis	Weighted NSTI	0.0234692827327
Ga51	Chronic_gastritis	Weighted NSTI	0.0284928431634
Ga52	Chronic_gastritis	Weighted NSTI	0.0232210470076
Ga53	Chronic_gastritis	Weighted NSTI	0.0241237951842
Ga54	Chronic_gastritis	Weighted NSTI	0.0249728754939
Ga55	Chronic_gastritis	Weighted NSTI	0.0245525155737
Ga56	Chronic_gastritis	Weighted NSTI	0.0247968261668
Ga57	Chronic_gastritis	Weighted NSTI	0.0220259722126
Ga58	Chronic_gastritis	Weighted NSTI	0.0235104559057
Ga59	Chronic_gastritis	Weighted NSTI	0.0340624095354
Ga60	Chronic_gastritis	Weighted NSTI	0.0248388022169
Ga61	Chronic_gastritis	Weighted NSTI	0.0371165514265
Ga62	Chronic_gastritis	Weighted NSTI	0.0315506630709
Ga63	Chronic_gastritis	Weighted NSTI	0.0190374080374
Ga64	Chronic_gastritis	Weighted NSTI	0.0255534901151

Ga65	Chronic_gastritis	Weighted NSTI	0.0283541207743
Ga66	Chronic_gastritis	Weighted NSTI	0.0243016108554
Ga67	Chronic_gastritis	Weighted NSTI	0.0251248866666
Ga68	Chronic_gastritis	Weighted NSTI	0.0329422136093
Ga69	Chronic_gastritis	Weighted NSTI	0.0234692839506
Ga70	Chronic_gastritis	Weighted NSTI	0.031955855137
Ga71	Chronic_gastritis	Weighted NSTI	0.0231236853239
Ga72	Chronic_gastritis	Weighted NSTI	0.0316024332108
Ga73	Chronic_gastritis	Weighted NSTI	0.0290991240903
Ga74	Chronic_gastritis	Weighted NSTI	0.0228351266279
Ga75	Chronic_gastritis	Weighted NSTI	0.0244748764283
Ga76	Chronic_gastritis	Weighted NSTI	0.0233080136803
Ga77	Chronic_gastritis	Weighted NSTI	0.0232002651225
Ga78	Chronic_gastritis	Weighted NSTI	0.0236748666552
Ga79	Chronic_gastritis	Weighted NSTI	0.0408731774542
Ga80	Chronic_gastritis	Weighted NSTI	0.0233605857388
Ga81	Chronic_gastritis	Weighted NSTI	0.0286796217287

SUPPLEMENTARY REFERENCES

1 van Doorn LJ, Figueiredo C, Rossau R, et al. Typing of Helicobacter pylori vacA gene and detection of cagA gene by PCR and reverse hybridization. *J Clin Microbiol* 1998;36:1271-6.
2 DeSantis TZ, Hugenholtz P, Larsen N, et al. Greengenes, a chimera-checked 16S rRNA gene database and workbench compatible with ARB. *Applied & Environmental Microbiology* 2006;72:5069-72.
3 Gevers D, Kugathasan S, Denson LA, et al. The treatment-naive microbiome in new-onset Crohn's disease. *Cell Host Microbe* 2014;15:382-92.
4 Langille MG, Zaneveld J, Caporaso JG, et al. Predictive functional profiling of microbial communities using 16S rRNA marker gene sequences. *Nat Biotechnol* 2013;31:814-21.
5 Parks DH, Tyson GW, Hugenholtz P, et al. STAMP: statistical analysis of taxonomic and functional profiles. *Bioinformatics* 2014;30:3123-4.
6 White JR, Nagarajan N, Pop M. Statistical methods for detecting differentially abundant features in clinical metagenomic samples. *PLoS Comput Biol* 2009;5:e1000352.
7 Walters WA, Caporaso JG, Lauber CL, et al. PrimerProspector: de novo design and taxonomic analysis of barcoded polymerase chain reaction primers. *Bioinformatics* 2011;27:1159-61.
8 Rinttila T, Kassinen A, Malinen E, et al. Development of an extensive set of 16S rDNA-targeted primers for quantification of pathogenic and indigenous bacteria in faecal samples by real-time PCR. *J Appl Microbiol* 2004;97:1166-77.
9 Horz HP, Vianna ME, Gomes BP, et al. Evaluation of universal probes and primer sets for assessing total bacterial load in clinical samples: general implications and practical use in endodontic antimicrobial therapy. *J Clin Microbiol* 2005;43:5332-7.
